# SIRT7-mediated NRF2 deacetylation promotes antioxidant response and protects against chemodrug-induced liver injury

**DOI:** 10.1038/s41419-025-07549-5

**Published:** 2025-04-01

**Authors:** Tingzi Yu, Cong Ding, Jinying Peng, Gaoshuang Liang, Yongyi Tang, Jinqiu Zhao, Zhuan Li

**Affiliations:** 1 The Key Laboratory of Study and Discovery of Small Targeted Molecules of Hunan Province, The Key Laboratory of Model Animals and Stem Cell Biology of Hunan Province, Engineering Research Center of Reproduction and Translational Medicine of Hunan Province, and Institute of Interdisciplinary Studies, Hunan Normal University School of Pharmaceutical Science, Changsha, Hunan China; 2 Department of infectious disease, The First Affiliated Hospital of Chongqing Medical University, Chongqing, China; 3 Department of Cancer center, University of Hawaii at Manoa, Honolulu, HI USA

**Keywords:** Apoptosis, Acetylation

## Abstract

NRF2 has been recognized as a central hub that neutralizes ROS and restores intracellular redox balance. In addition to KEAP1 mediated ubiquitin-proteasome degradation, post-translational modifications of NRF2 are critical for regulating its nuclear translocation and activation but precise mechanisms underly this regulation remain elusive. In this study, we found that SIRT7 was sufficient and essential for NRF2 nuclear localization and activation. Knockdown of SIRT7 significantly impaired intercellular ROS homeostasis and increased apoptosis in response to oxidative stress including chemodrug treatment. SIRT7 interacted with NRF2 and induced its deacetylation, by which inhibited binding of NRF2 to KEAP1, enhanced NRF2 protein stability and promoted its nuclear translocation. SIRT7 induced NRF2 deacetylation at K443 and K518 sites. Lysine-arginine mutations of these sites (2KR NRF2) significantly reduced KEAP1/NRF2 binding, increased NRF2 nuclear translocation and target gene expression, decreased intercellular ROS level, whereas lysine-glutamine (2KQ) mutant showed similar subcellular localization and functions with WT. Knockdown SIRT7 in hepatocyte exacerbated Oxaliplatin (Oxa) induced hepatic injury and inflammation. While AAV8-NRF2-mediated hepatic NRF2 overexpression or NRF2 agonist significantly prevented Oxa-induced elevation of ALT levels, sinusoidal dilatation and inflammation in *SIRT7*^*HKO*^ mice. Our data thus uncovered previously unidentified role of SIRT7 in modulating NRF2 nuclear localization and activation via deacetylation. Activating SIRT7 might offer protection against chemodrug-induced liver injury.

## Introduction

Reactive oxygen species (ROS) are generated during intracellular oxidative metabolism by mitochondria as well as through exogenous stimulus such as cytokines and bacterial infections [1, 2]. Cells normally counteract the detrimental effects of ROS and electrophiles through the activation of transcription factor nuclear factor-E2-related factor 2 (NRF2) which has been recognized as a central hub that neutralizes ROS and restores intracellular redox balance [3–5]. In homeostatic status, NRF2 locates in the cytoplasm where it is rapidly degraded by kelch-like ECH-associated protein 1 (KEAP1) mediated ubiquitin-proteasome degradation [6]. In response to different activating stimuli, KEAP1 is modified at several key reactive cysteine residues [7] which results in conformational changes [8] and impairs its ability to target NRF2 for ubiquitination and degradation. Consequently, NRF2 is stabilized and translocate into nuclear where it binds to the conserved antioxidant response element (ARE) at the promoter regions of a battery of antioxidative and cellular defense targets, such as NADPH quinone oxidoreductase 1 (NQO1) [9], heme oxygenase-1 (HO-1) [10], and glutamate cysteine ligase modifier subunit (GCLM) [11], and then elicits robust anti-toxification responses [12, 13]. Targeting KEAP1 has been proposed as option to activate NRF2 mediated antioxidative response, but the upstream modulator of KEAP1 and selectivity of covalent cysteine modification by the molecules remain largely unclear.

In addition to the negative regulator KEAP1, nuclear localization and activation of NRF2 are also regulated by post-translational modifications (PTMs) including phosphorylation, ubiquitination, acetylation, and sumoylation [14–17]. Among those PTMS, ubiquitination primarily regulates NRF2 stability through the KEAP1-mediated degradation pathway [18], while acetylation affects its expression and activation. Lysine acetylation is involved in the regulation of multiple key cellular processes including gene transcription, DNA damage repair, cell division, and signal transduction [19, 20]. Multiple acetylation sites including lysine 506, 508, 588, and 591 have been identified within NRF2 DNA binding domains and regulate its transcription activity [21–23]. Emerging findings indicate that NRF2 acetylation augments its promoter-specific DNA binding ability of NRF2 and thus modulation of NRF2 lysine acetylation might serve as a novel regulatory mechanism in the NRF2-dependent antioxidant response [22]. However, the precise mechanisms underlie how acetylation modulates NRF2 nuclear localization and activation, particularly in antioxidative response remain poorly understood.

SIRT7 is a family member of NAD^+^-dependent histone deacetylases, which is the only SIRTs protein member highly enriched in nucleolar compartments [24, 25]. In addition to histone protein, SIRT7 is confirmed to target several non-histone proteins including p53, GABP-β, FOXO3, and U3-55k for deacetylation and has been implicated in multiple cellular functions including hepatic lipid metabolism, mitochondrial homeostasis, and adipogenesis [26–29]. SIRT7 plays a significant role in the progression of fatty liver disease [30], liver fibrosis [31] and liver cancer [32]. SIRT7 directly targets HMGB1 and participates in regulating the expression and distribution of HMGB1, thus affecting the autophagy and activation level of hepatic stellate cells [31]. Emerging evidence suggests that SIRT7 is a dynamic regulator of oxidative stress through various mechanisms including the regulation of ROS production [33] and mitochondrial function [27] as well as promotion of autophagy [34]. However, downstream targets that are responsible for SIRT7 mediated antioxidative response remain elusive.

In the present study, we revealed that SIRT7 directly interacted with NRF2 and induced NRF2 deacetylation at K443 and K518 which is crucial for NRF2 nuclear translocation and activation. Knockdown of SIRT7 resulted in a significant increase of impairment of antioxidative response and cell apoptosis in response to oxidative stress. Knockdown SIRT7 in hepatocyte exacerbated Oxa induced hepatic injury and inflammation while NRF2 agonist or AAV8-NRF2 significantly reversed Oxa-induced liver injury in *SIRT7*^*HKO*^ mice. Our data thus uncovered previously unidentified role of SIRT7 in modulating NRF2 nuclear localization and activation via deacetylation and protecting chemodrug-induced liver injury.

## Materials and methods

### Animals and cell culture

Male C57BL/6 mice (4–6 weeks) were obtained from Hunan Silaike Jingda Laboratory Animal Co., Ltd. (Hunan, China). *SIRT7*^*flox/flox*^ and Albumin Cre mice were purchased from Shanghai Model Organisms Center (Shanghai, China). Heterozygotes were bred to obtain both hepatocyte specific SIRT7 knockout (*SIRT7*^*HKO*^) and wild-type (WT) littermates. All mice were housed in a temperature-controlled, specific pathogen-free environment with 12-h light-dark cycles. All animal handling procedures were approved by the Institutional Animal Care and Use Committees at Hunan Normal University School of Medicine. Mice were intraperitoneally injected with Oxaliplatin dissolved in 5% glucose solution (Targetmol, China), 4 mg/kg, or injected with 5% glucose solution (Kelun, China) as control once a day for 12 days. *SIRT7*^*HKO*^ mice were randomly divided into 2 groups, with 6–8 mice per group. Adeno-associated virus (AAV8)-TBG NRF2 vectors (Hanbio, China) were used to overexpress NRF2 in the livers of *SIRT7*^*HKO*^ mice. One week before Oxaliplatin treatment, AAV8-TBG NRF2 vectors were injected into one group of mice via tail vein injection, while the other group received injections of control virus vectors. Then, both groups were given Oxaliplatin treatment at equal doses and durations. In parallel, *SIRT7*^*HKO*^ mice were randomly divided into 2 groups, with 6-8 mice per group. Oltipraz (MedChemExpress, New Jersey, USA) was dissolved in DMSO, followed by the addition of PEG300 and sterile saline. After one week of Oxaliplatin administration, one group was orally administered 100 mg/kg Oltipraz for 5 days, while the other group received a corresponding volume of solvent based on body weight. Mice were fasted overnight prior to sacrifice and serum samples and liver tissues were harvested. Liver tissue was fixed with 10% formaldehyde solution or storaged in liquid nitrogen for further analysis. The cells were maintained in DMEM (Gibco, USA), supplemented with 10% fetal bovine serum (Gibco, USA) and antibiotic-antifungal substance (Basal Media, Shanghai, China) and incubated at 37 °C in humidified air containing 5% CO_2_. The cell lines used in this study were obtained from icell (Shanghai, China). They were recently authenticated using STR profiling and tested for mycoplasma contamination to ensure validity and purity.

### Antibodies and chemicals

Anti-F4/80(#70076), anti-SIRT7(#5360), anti-SIRT3(#5490), anti-SIRT4(#69786), anti-PARP(#9542) and anti-Bcl2(#15071) were purchased from Cell Signaling Technology (Danver, USA), anti-cleaved-caspase3(#82707-13-RR), anti-NRF2(#16396-1-AP) and anti-KEAP1(#10503-2-AP) were purchased from Proteintech (Wuhan, China), anti-GAPDH(#41549) was purchased from Signalway Antibody (Maryland, USA), anti-acetylation K(#BS74032) was purchased from Bioworld Technology (Minnesota, USA). Oxaliplatin, Oltipraz and Rapamycin were purchased from Targetmol (Shanghai, China) and MedChemExpress (New Jersey, USA), respectively. Adeno-associated virus (AAV8)-TBG NRF2 and control virus vectors were purchased from Hanbio (Shanghai, China).

### Reactive oxygen species measurement

Intracellular ROS and superoxide anion levels were measured by using DCFH-DA (Beyotime, Cat#S0033S) [35] and dihydroethidium (DHE) (APExBio, Cat#C3807) [36], respectively. Mitochondrial ROS was measured using MitoSOX Red (MedChemExpress, Cat#HY-D1055) [37]. Briefly, 2.5 × 10^5^ cells were seeded into each well of 12-well plates and cultured overnight. The next day, cells were treated with Oxaliplatin for 24 h, followed by adding probes into the culture medium for 30 min at 37 °C. After washing with PBS, cells were photographed under fluorescence microscope (Leica Biosystems, Germany). All data were obtained by randomly selecting several areas and summarized as mean ± SD from each experiment.

### Biochemical assays

ALT and GSH activity were determined using an ALT reagent kit (Nanjing JianCheng Bioengineering Institute, China), and Triglyceride levels were measured by a Triglyceride (TG) level measurement kit (Beijing Boxbio Science & Technology, China).

### RNA isolation and real-time quantitative PCR

Total RNA was isolated from cells by using the Trizol reagent (Vazyme Biotech, China), followed by cDNA synthesis using an RNA reverse transcription kit (Vazyme Biotech, China). Quantification was conducted by qPCR SuperMix (TransGen Biotech, China) according to manufacturer’s protocol. The results were calculated with the 2^−ΔΔCt^ method, with GAPDH serving as the internal reference. The primers sequences are shown in Table 1 and Table 2.

**Table 1:** Primer (Mouse) sequences used for RT-qPCR.

Primer	Forward (5′-3′)	Reverse (5′-3′)
SIRT7	CTGGAGATTCCTGTCTACAACCG	AGTGACTTCCTACTGTGGCTGC
NRF2	ACAGTGCTCCTAT GCGTGAA	GAGCCTCTAAGCGGCTTGAA
KEAP1	CGGGGACGCAGTGATGTATG	TGTGTAGCTGAAGGTTCGGTTA
HO-1	TGCTAGCCTGGTGCAAGATA	GCCAACAGGAAGCTGAGAGT
NQO1	CAGCTCACCGAGAGCCTAGT	ACCACCTCCCATCCTTTCTT
GAPDH	CGTCCCGTAGACAAAATGGT	TTGAGGTCAATGAAGGGGTC
IL-6	TTCCATCCAGTTGCCTTCTT	CAGAATTGCCATTGCACAAC
TNF-a	AGGCTCTGGAGAACAGCACAT	TGGCTTCTCTTCCTGCACCAAA

**Table 2:** Primer (Human) sequences used for RT-qPCR.

Primer	Forward (5′-3′)	Reverse (5′-3′)
SIRT7	ATGAGCAGAAGCTGGTGC	CTGTCTGGTGTCTGTGGA
NRF2	TACTCCCAGGTTGCCCACA	CATCTACAAACGGGAATGTCTGC
KEAP1	AGAGGTGGTGGTGTTGCTTAT	TGGAGATGGAGGCCGTGTA
HO-1	AAGATTGCCCAGAAAGCCCTGGAC	AACTGTCGCCACCAGAAAGCTGAG
NQO1	GAAGAGCACTGATCGTACTGGC	GGATACTGAAAGTTCGCAGGG
GCLM	TGTCTTGGAATGCACTGTATCTC	CCCAGTAAGGCTGTAAATGCTC
GAPDH	AGGGCTGCTTTTAACTCTGGT	CCCCACTTGATTTTGGAGGGA
P53	CCTCAGCATCTTATCCGAGTGG	TGGATGGTGGTACAGTCAGAGC
LC3	CGCTACAAGGGTGAGAAGC	TGGACACACTCACCATGCT
Beclin1	GAGAACCTCAGCCGAAGACT	CCTCTAGTGCCAGCTCCTTT
p62	AGAACAAGTACCTGCCCGAA	TGGTCCAAGAATCTTCCCCA
Atg5	GGACAGTTGCACACACTAGG	TCCGGGTAGCTCAGATGTTC
mTOR	CTGAGCAGAACCAGGGTACA	GGACACAGCTGGGTAGAACT

### Immunohistochemistry Staining (IHC)

IHC on formalin-fixed sections was performed by deparaffinization and rehydration, followed by antigen retrieval by heating in a pressure cooker (121 °C) for 5 min in EDTA (1 mmol/L) or sodium citrate solution (2.94 g/L). After blocking with 4% BSA at room temperature for 1 h, liver slices were incubated with primary antibodies overnight at 4 °C. After washing with PBS/PBST (0.1% Tween-20), incubated the slices with the secondary antibodies at room temperature for 30 min, followed by washing with PBS/PBST and adding DAB chromogenic solution (Zhong Shan-Golden Bridge Biological Technology, China). Then, counterstain with hematoxylin. The slices were sealed with neutral gum and images were acquired by using a Zeiss Axiolab 5 Digital Lab Microscope (Carl Zeiss AG, Germany).

### Western blotting analysis

Briefly, total proteins were isolated from the liver tissues. Protein concentrations were determined using the BCA protein assay kit (Beyotime Biotechnology, China). Equal amounts of proteins were loaded onto an SDS-polyacrylamide gel for electrophoresis and then transferred onto a polyvinylidene-difluoride (PVDF) membrane using a transblotting apparatus. The blots were immersed in blocking solutions (TBST containing 5% skim milk) for 1 h at room temperature. The blots were then incubated with the primary antibodies of anti-acetylation K (1:1000), anti-SIRT7 (1:1000), anti-SIRT3 (1:1000), SIRT4 (1:1000), anti-NRF2 (1:5000), anti-KEAP1 (1:4000), anti-PARP (1:1000), anti-Bcl2 (1:1000) and anti-GAPDH (1:10000) at 4 °C overnight. The next day, the membranes were washed and incubated with the corresponding secondary antibodies (1:3000) for 1 h at room temperature. Protein bands were detected using an enhanced chemiluminescent substrate. The intensity of each band was quantified by using Image J (National Institute of Health, USA).

### Isolation of primary mouse hepatocytes

Primary Mouse Hepatocytes (PMH) were isolated and purified from C57BL/6, WT, or *SIRT7*^*HKO*^ mice using collagenase and cultured in DMEM. The liver was perfused with a buffer saline solution, followed by digestion with collagenase, and primary hepatocytes were collected by centrifugation at 50 *g*. DMEM medium was added and then cultured in incubator for 2 h, DMEM medium was replaced based on the adherence situation, and the cells were ready for subsequent experiments after overnight culture.

### Cell transfection

Flag-SIRT7 and Flag-SIRT7-H187Y (Flag-SIRT7 HY) plasmids were respectively provided by Drs. Eric Verdin and Katrin Chua via Addgene (Cambridge, MA). Flag-NRF2 plasmid was purchased from iGeneBio (Guangzhou, China). NRF2 KR and KQ mutations were generated by Q5 Site-Directed Mutagenesis Kit (New England biolabs, Ipswich, MA). Transfection of plasmids was performed in the serum-free medium by using Lipofectamine 6000 (Beyotime, China) according to the manufacturer’s instructions.

### Lentiviral packaging and infection

Lentiviral packaging was used to generate stable SIRT7 knockdown cells. Briefly, HEK293T cells were co-transfected with viral vectors and packaging plasmids. 48 h after transfection, the progeny viruses released from HEK293T cells were filtered through a 0.45 μm filter, and collected to infect cells of interest. 36–48 h after infection, Huh7 or HEK293T cells were selected with 2 μg/ml puromycin in DMEM. The medium that contained puromycin was changed every 2–3 days.

### Co-immunoprecipitation (Co-IP) and Ubiquitination assay

Cells were cultured and treated with or without Oxaliplatin solution for 24 h. The protein was extracted and 400 μg of total protein were used for co-immunoprecipitation (Co-IP) by using Biolinkedin Basic Co-IP kit (Biolinkedin, China) and the immunocomplex was analyzed by Western blot. For the ubiquitination assay, cells were treated with MG132 (50 μM) for 4 h before harvest. The protein was purified using a Co-IP kit, and ubiquitination levels were evaluated by Western blot.

### TUNEL staining

Cell samples were fixed with 4% paraformaldehyde and permeabilized by 0.2% TritonX-100, after washing with PBS, TUNEL assay was performed by TUNEL staining Kit (Vazyme Biotech, China). In brief, TUNEL detection solution was added dropwise and incubated at 37 °C for 60 min. Cells were washed 3 times with PBS and DAPI staining solution was added and incubated at room temperature for 5 min. After PBS wash, cells were sealed with anti-fluorescence quenching solution, and photographed under fluorescence microscope (Leica Biosystems, Germany).

### Immunofluorescence (IF)

Cells were seeded on glass coverslips and fixed in 4% paraformaldehyde solution. After washing with PBS, 0.2% Triton X-100 was used to make cells permeable. Following a wash with PBS again, cells were incubated for 1 h with 4% BSA. The primary antibody was used to incubate cells at room temperature for 1 h. After washing three times with PBS, the corresponding secondary antibody was added to glass and incubated for 1 h in the dark at room temperature. Coverslips were additionally incubated with DAPI for 5 min at room temperature to stain nuclear DNA. Images were acquired using a confocal fluorescence microscope (Leica Biosystems, Germany).

### Statistical analysis

Statistical analysis of experimental results was performed by using GraphPad Prism 9. All experiments were repeated at least three times, independently, and data were presented as mean ± SD. One-way ANOVA was used for the comparison of means between multiple groups, and the Tukey test was used for two-way comparison within groups. The variance between groups satisfied the assumptions required for the appropriate test. Unless otherwise stated, a *p* < 0.05 was considered statistically significant. No blinding was performed during the experiment and outcome assessments.

## Results

### SIRT7 was sufficient and essential for NRF2 expression and nuclear translocation

**Fig. 1:**
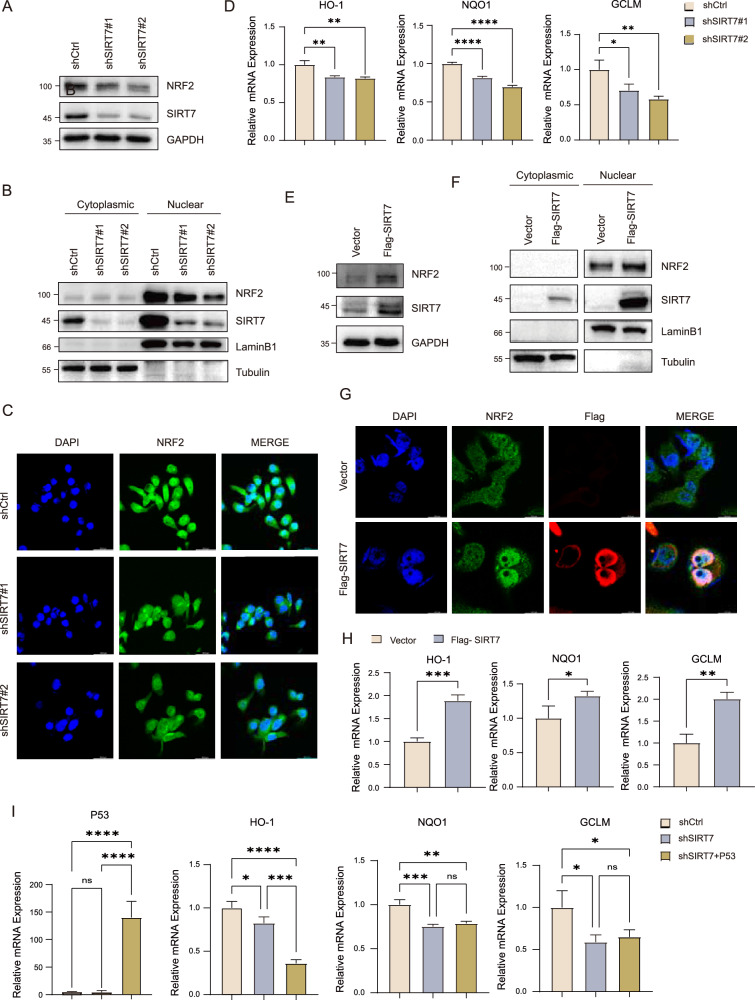
SIRT7 was sufficient and essential for NRF2 expression and nuclear translocation. **A** Protein level of NRF2 was determined by western blotting in WT and SIRT7-KD Huh7 cells. **B** Relative amounts of NRF2 in the nuclear or cytoplasmic fractions were determined by a cell fraction assay in SIRT7-KD Huh7 cells. **C** Representative immunofluorescence stains of NRF2 (green) incorporation with DAPI counterstain (blue) in WT and SIRT7-KD Huh7 cell. Scale bar, 36.8 μm. *n* = 5. **D** Relative mRNA expression of NRF2 target genes were determined by RT-qPCR in WT and SIRT7-KD Huh7 cells. **E** Western blotting analysis of NRF2 protein level was performed in Huh7 cells stably overexpressing either vector control or SIRT7. **F** Relative amounts of NRF2 in the nuclear or cytoplasmic fractions were determined by a cell fraction assay after SIRT7 overexpression. **G** Immunofluorescence staining of NRF2 (green) in Huh7 cells transfected with FLAG-tagged SIRT7 (red). **H** Relative mRNA expression of NRF2 target genes were determined by RT-qPCR in Huh7 cells stably overexpressing SIRT7. **I** SIRT7-KD Huh7 cells were transfected with HA-tagged P53. Relative mRNA expression of NRF2 target genes were determined by RT-qPCR. Data derived from three independent experiments were presented as mean ± SEM. *p < 0.05, **p < 0.01, ***p < 0.001, ****p < 0.0001.

To assess potential role of SIRT7 on NRF2 activity, we knockdown SIRT7 by shRNA (shSIRT7) in Huh7 cells and examined NRF2 protein expression, nuclear translocation and activity (Fig. 1A–D). Knockdown of SIRT7 markedly decreased NRF2 protein expression when compared to control cells (shCtrl, Fig. 1A). Subcellular fractionation and immunofluorescence further indicated that knockdown of SIRT7 significantly reduced the nuclear localization of NRF2 (Fig. 1B, C). Additionally, knockdown of SIRT7 significantly decreased the expression of NRF2 target genes including HO-1, NQO1, and GCLM (Fig. 1D). In contrast, overexpression of SIRT7 in Huh7 cells dramatically increased NRF2 protein expression and nuclear translocation (Fig. 1E–G). The mRNA expression of NRF2 target genes was also significantly elevated by SIRT7 overexpression (Fig. 1H). Since p53 counteracts the NRF2-induced transcription [38] and we have previously reported that SIRT7 modulates p53 activity [39] and thus determined whether p53 participated in SIRT7 mediated NRF2 regulation and the results indicated that overexpression of p53 in SIRT7-KD cells barely impacted expression of NRF2 target genes (Fig. 1I).

### SIRT7 was essential for ROS homeostasis and cell survival under oxidative stress

**Fig. 2:**
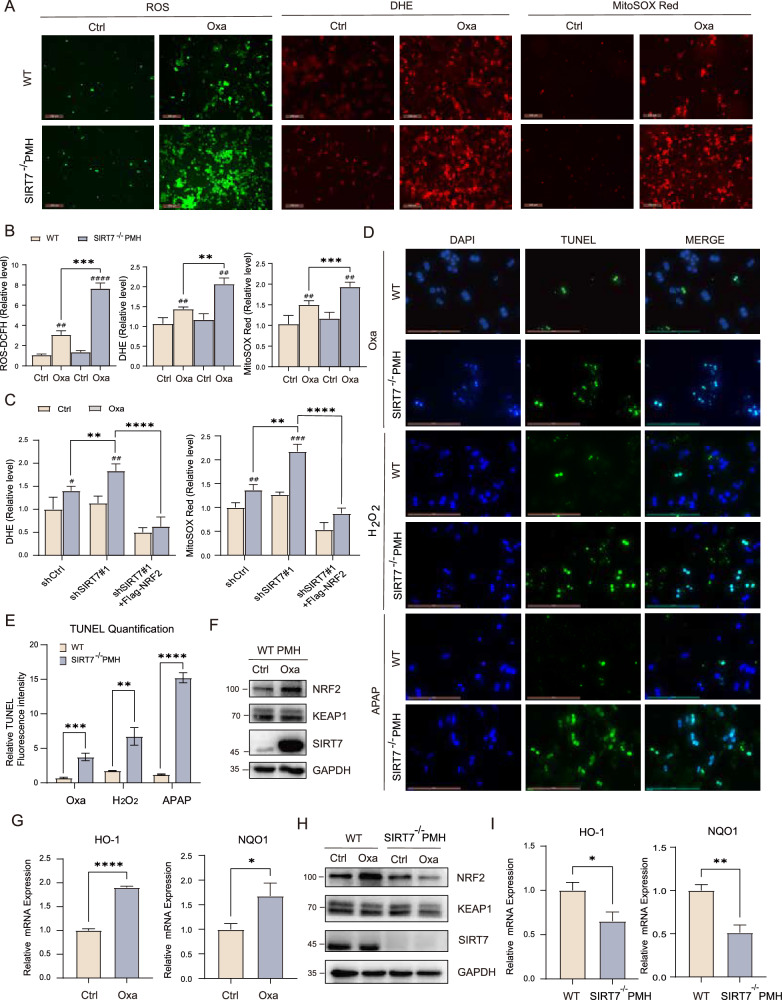
SIRT7 was essential for ROS homeostasis and cell survival under oxidative stress. **A** PMH from SIRT7^HKO^ and their WT littermates were incubated with control or Oxaliplatin medium (15 μM) for 24 h and then stained with DCFH-DA, 5 μM DHE (dihydroethidium) and 5 μM MitoSOX Red respectively, cells were briefly washed with PBS and the fluorescence signal was recorded using Leica confocal microscope and quantified using Image J. Scale bar, 200 μm. *n* = 5. **B** Quantitative analysis of relative fluorescence intensity in (**A**). **C** Huh7 cells were transfected with shCtrl, sh-SIRT7 or sh-SIRT7 + FLAG-tagged NRF2. The DHE and MitoSOX Red fluorescence values in Oxaliplatin (15 μM, 24 h) or control-treated Huh7 cells above were analyzed. Relative levels were respectively quantified. Scale bar, 200 μm. *n* = 5. **D** Representative images of TUNEL staining of primary mouse hepatocytes with or without SIRT7 treated with Oxaliplatin 15 μM for 24 h, H_2_O_2_ 400 μM for 3 h or APAP 20 μM for 24 h. Scale bar, 100 μm. *n* = 5. **E** Quantitative analysis of relative fluorescence intensity in (**D**). **F** Protein level of NRF2/KEAP1 pathway was evaluated by western blotting in primary mouse hepatocyte with or without Oxaliplatin treatment. **G** RT-qPCR analysis of HO-1 and NQO1 mRNA levels was performed in primary mouse hepatocyte after Oxaliplatin treatment. **H** Protein level of NRF2/KEAP1 pathway were evaluated by western blotting in primary mouse hepatocyte isolated from WT and *SIRT7*^*HKO*^ mice after Oxaliplatin treatment. **I** RT-qPCR analysis of HO-1 and NQO1 mRNA levels was performed in the above primary mouse hepatocyte. Data derived from three independent experiments were presented as mean ± SEM. ^#^*p* < 0.05, ^##^*p* < 0.01, ^###^*p* < 0.001, ^####^*p* < 0.0001 compared to Scramble group, and *p < 0.05, **p < 0.01, ***p < 0.001, ****p < 0.0001 compared to Scramble group or the indicated two groups.

Since NRF2 is one of the main mechanisms that responsible for antioxidative response, we thus questioned whether SIRT7 participated in regulations of ROS homeostasis and antioxidative response in PMH. Knockout SIRT7 (SIRT7^−/−^ PMH) hardly enhanced Intracellular ROS, superoxide anion as well as mitochondrial ROS levels as evidenced by intercellular ROS, DHE, and MitoSOX Red (Fig. 2A, B). We further treated cells with Oxa that cause ROS accumulation and the results indicated that Oxa treatment significantly promoted ROS accumulation in wild type (WT) cells, and this was further enhanced in SIRT7^−/−^ PMH (Fig. 2A, B). Similar results were observed by using SIRT7 knockdown cell and we further confirmed that restoring NRF2 expression was sufficient to prevent elevation of Oxa-induced ROS accumulation in SIRT7 knockdown cells (Fig. 2C). Above data indicated that SIRT7 was responsible for NRF2 mediated ROS homeostasis, we thus further questioned whether SIRT7 regulates cell survival in response to oxidative stress. We isolated PMH and treated them with varies reagents that cause ROS accumulation and cell death including Oxa, hydrogen peroxidase (H_2_O_2_) and acetaminophen (APAP) and measured cell death by TUNEL assay. In all cases, knockout of SIRT7 in PMH significantly enhanced TUNEL positivity compared to WT cells (Fig. 2D, E). In addition, we found that Oxa treatment significantly increased NRF2 protein expression and target gene expression in vitro (Fig. 2F, G). Knockout of SIRT7 markedly impaired Oxa-induced NRF2 upregulation and target genes expression in PMH cells (Fig. 2H, I). In contrast, overexpression of SIRT7 sufficiently prevented Oxa induced ROS accumulation and cell death in vitro, while knockdown NRF2 reversed these effects mediated by SIRT7 (Fig. S1A, S1B). Since NRF2 and autophagy exist crosstalk [40] and we thus examined whether SIRT7 regulated cell survival through autophagy. SIRT7 knockdown did not affect the mRNA levels of autophagy-related gene (Fig. S2A), while markedly decreased the protein level of p62 and LC3 (Fig. S2B, S2C). To further confirm whether autophagy have potential role in SIRT7 mediated cell survival, we used autophagy activator rapamycin in SIRT7 KD Huh7 cells before Oxa treatment. Knockdown of SIRT7 significantly increased Oxa induced cell death but rapamycin nearly had no effects of preventing cell death in these cells (Fig. S2D, S2E).

### SIRT7 interacted with and deacetylated NRF2

**Fig. 3:**
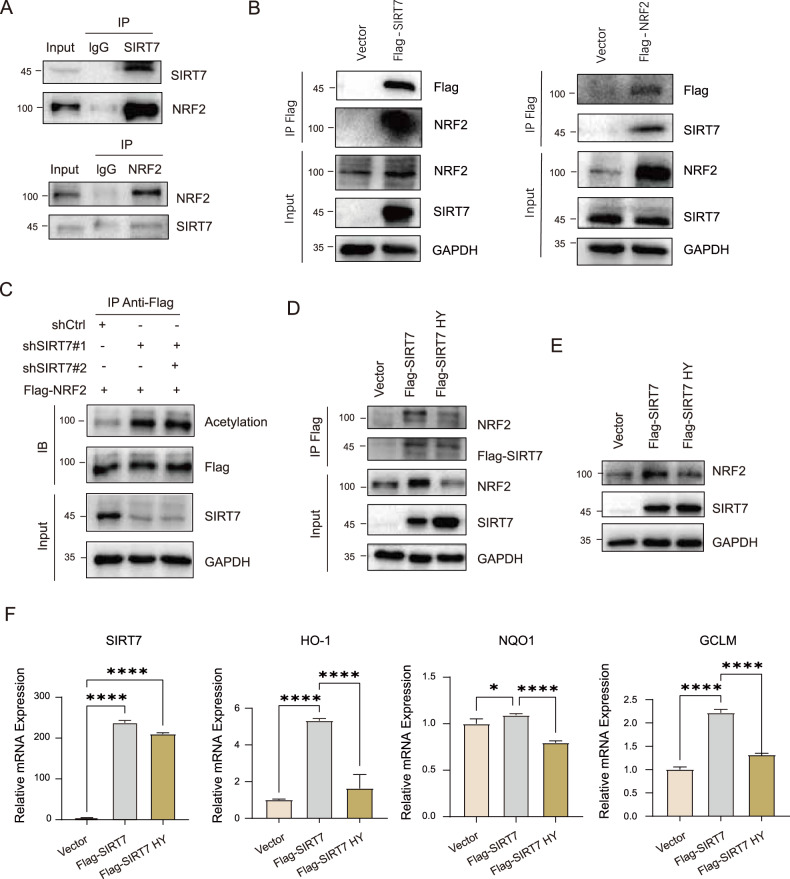
SIRT7 interacted with and deacetylated NRF2. **A** Co-IP experiment of SIRT7 with NRF2 was performed in mouse primary hepatocytes. **B** Co-IP experiment of FLAG-tagged SIRT7 with NRF2 and FLAG-tagged NRF2 with SIRT7 was performed in Huh7 cells. **C** Deacetylation of NRF2 by SIRT7 in cells. WT and SIRT7-KD HEK293T cells were transfected with FLAG-tagged NRF2 for 42 h and then incubated in the presence of TSA (5 μM) for 2 h. Cells were lysed and cell extracts were subjected to immunoprecipitation with FLAG-tagged beads. Acetylated NRF2 was analyzed by western blotting with anti-acetylation and total protein was evaluated in whole cell lysates (Input). **D** Co-IP experiment of FLAG-tagged SIRT7 or FLAG-tagged SIRT7 HY with NRF2 was performed in Huh7 cells. **E** Protein level of NRF2 was determined by western blotting in Huh7 cells stably overexpressing SIRT7 WT or HY mutant. **F** Relative mRNA expression of NRF2 target genes were determined by RT-qPCR in Huh7 cells stably overexpressing FLAG-tagged SIRT7 or FLAG-tagged SIRT7 HY mutant. Data derived from three independent experiments were presented as mean ± SEM. *p < 0.05, **p < 0.01, ***p < 0.001, ****p < 0.0001.

To investigate how SIRT7 regulates NRF2 protein expression and activity. We assessed protein-protein interactions by using coimmunoprecipitation (Co-IP) and the results indicated that both endogenous and exogenous SIRT7 interacted with NRF2 (Fig. 3A, B). To further examine whether SIRT7 deacetylases NRF2, we knocked down SIRT7 and evaluated the acetylation level of NRF2. Knockdown of SIRT7 resulted in the increased deacetylation level of NRF2 (Fig. 3C). To further evaluate the role of SIRT7 dependent deacetylation on NRF2 protein expression and activation, we used enzymatic inactive SIRT7 H133Y (SIRT7 HY). SIRT7 HY similarly interacted with NRF2 but barely affected NRF2 protein expression (Fig. 3D, E). More importantly, SIRT7 dependent NRF2 target gene expression was almost completely abolished by SIRT7 HY mutation (Fig. 3F).

### SIRT7 inhibited ubiquitin-proteasome degradation of NRF2

**Fig. 4:**
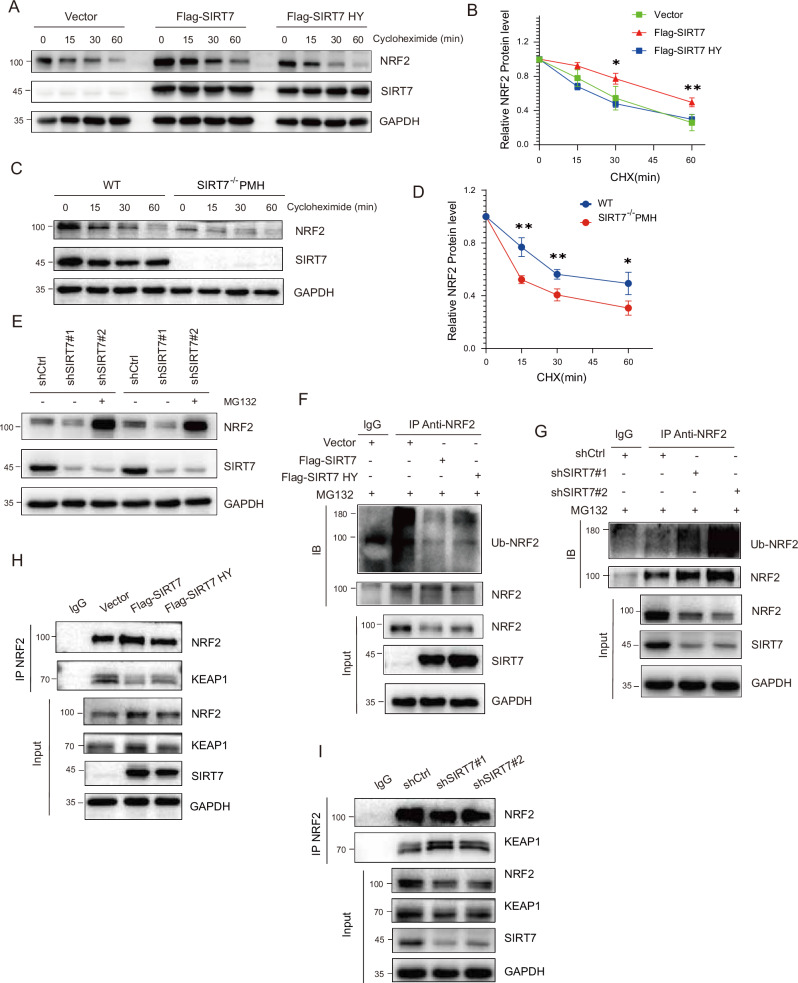
SIRT7 inhibited ubiquitin-proteasome degradation of NRF2. **A** Huh7 cells were transfected with plasmid as indicated. CHX (100 μg/mL) was added, and cells were harvested at the indicated time points. Protein level of endogenous NRF2 was determined by western blotting. **B** Quantitative analysis of NRF2 panels in (**A**). **C** WT and *SIRT7*^*HKO*^ mouse primary hepatocytes were treated with 100 μg/ml of CHX and harvested at the indicated time points. Protein level of NRF2 was determined by western blotting. **D** Quantitative analysis of NRF2 panels in (**C**). **E** NRF2 protein level were determined by western blotting in control and SIRT7 KD Huh7 cells in the presence or absence of proteasome inhibitor MG132. Ubiquitination-NRF2 (Ub-NRF2) was determined by immunoprecipitation (IP) of NRF2 with a subsequent WB assay with anti-ubiquitin antibody in SIRT7 overexpressed (**F**) or KD (**G**) Huh7 cells. The binding of NRF2 and KEAP1 were analyzed by western blotting in SIRT7 overexpressed (**H**) or KD (**I**) Huh7 cells. Data derived from three independent experiments were presented as mean ± SEM. *p < 0.05, **p < 0.01, ***p < 0.001, ****p < 0.0001.

We then examined how SIRT7 increased NRF2 expression and measured the half-life of endogenous NRF2 in the presence of SIRT7 or SIRT7 HY. The results indicated that half-life of NRF2 was remarkably increased by SIRT7 which was completely abolished by SIRT7 HY (Fig. 4A. B). Conversely, knockout of SIRT7 in PMH shortened the half-life of NRF2 (Fig. 4C, D). The decrease of NRF2 mediated by SIRT7 knockdown was reversed by MG132 treatment, but SIRT7 degradation was not prevented by MG132, which was consistent with the previous report [41] (Fig. 4E). We further evaluated whether SIRT7 protects NRF2 from proteasome-dependent degradation via inhibiting NRF2 ubiquitination. Overexpression of SIRT7 dramatically repressed NRF2 ubiquitination (Fig. 4F) while knockdown of SIRT7 showed opposite effects (Fig. 4G). However, overexpression of SIRT7 HY only partially reversed SIRT7 mediated repression of NRF2 ubiquitination (Fig. 4F). Since NRF2/KEAP1 interaction is critical in determining NRF2 ubiquitination, we thus questioned whether SIRT7 regulated NRF2 stability via modulating NRF2/KEAP1 interaction. The results indicated that overexpression of SIRT7 significantly interrupted NRF2/KEAP1 interaction while SIRT7 HY mildly affected NRF2/KEAP1 interaction, suggesting that deacetylation activity of SIRT7 sufficient but not essential for NRF2/KEAP1 interaction and NRF2 degradation (Fig. 4H). Consistently, knockdown of SIRT7 markedly increased NRF2/KEAP1 interaction (Fig. 4I).

### SIRT7 deacetylated NRF2 at Lysine 443 and Lysine 518

**Fig. 5:**
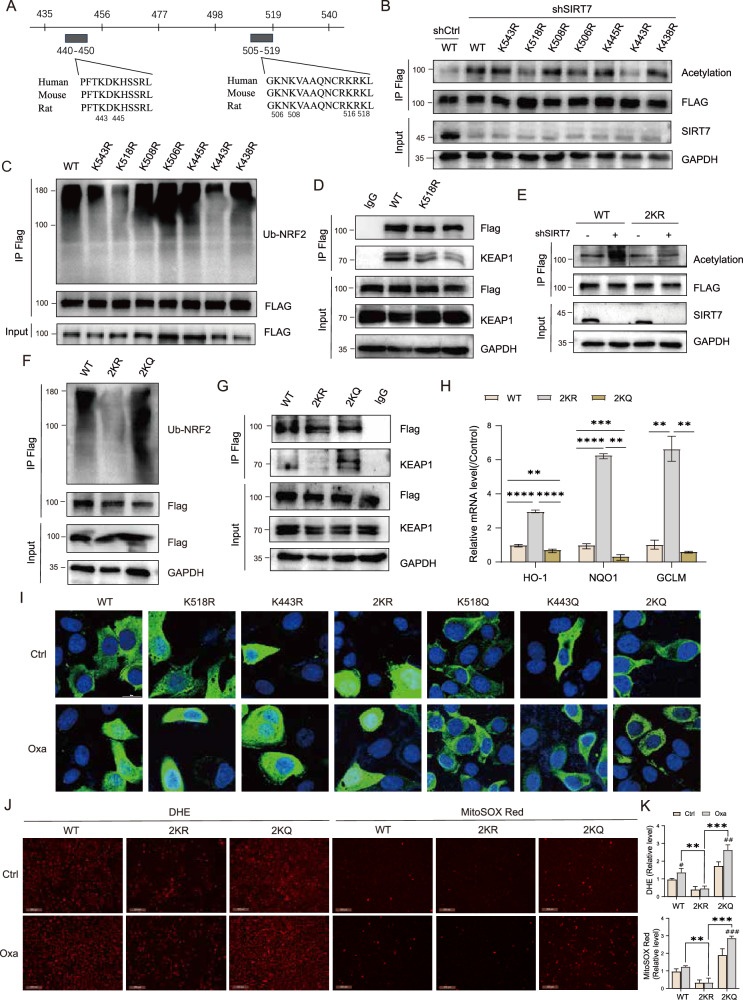
SIRT7 deacetylated NRF2 at Lysine 443 and Lysine 518. **A** Schematic illustration of the predict motif (435-540AA) in NRF2 by the Predict Protein database. **B** WT and SIRT7-KD HEK293T cells were transfected with wild type FLAG-tagged NRF2 (WT), FLAG-tagged NRF2 K543R, K518R, K508R, K506R, K445R, K443R, K438R for 42 h then incubated with TSA for 2 h. Acetylated NRF2 was analyzed by western blotting with anti-acetylation and total protein was evaluated in whole cell lysates (Input). **C** HEK293T cells were transfected with plasmid as indicated. Cells were treated with 50 μM of MG132 for 4 h. The ubiquitinated forms of exogenous NRF2 were analyzed by western blotting with ubiquitination antibody. **D** HEK293T cells were transfected with FLAG-tagged NRF2 K518R, K443R, then, Co-IP experiment of FLAG-tagged NRF2 with KEAP1 was performed. **E** WT and SIRT7-KD HEK293T cells were transfected with WT, FLAG-tagged NRF2 K443R, K518R, 2KR (K443R, K518R), acetylated NRF2 was analyzed as in (**B**). **F** HEK293T cells were transfected with plasmid as indicated, the ubiquitinated forms of NRF2 were analyzed as in (**C**). **G** HEK293T cells were transfected with FLAG-tagged NRF2 WT, 2KR, 2KQ (K443Q, K518Q), then, Co-IP experiment of FLAG-tagged NRF2 with KEAP1 was performed. **H** HEK293T cells were transfected with plasmid as indicated. Relative mRNA expression of NRF2 target genes were determined by RT-qPCR. **I** Immunofluorescence staining of NRF2 (green) in Huh7 cells transfected with FLAG-tagged NRF2 WT, K518R, K443R, 2KR, K518Q, K443Q, 2KQ with or without Oxaliplatin treatment. **J** Huh7 cells were transfected with FLAG-tagged NRF2 WT, 2KR, 2KQ, and the fluorescence signal was recorded using Leica confocal microscope. Scale bar, 200 μm. *n* = 5. **K** Quantitative analysis of relative fluorescence intensity in (**J**). Data derived from three independent experiments were presented as mean ± SEM. ^#^*p* < 0.05, ^##^*p* < 0.01, ^###^*p* < 0.001, ^####^*p* < 0.0001 compared to Scramble group, and *p < 0.05, **p < 0.01, ***p < 0.001, ****p < 0.0001 compared to Scramble group or the indicated two groups.

To further explore the detailed mechanism underlying SIRT7-dependent NRF2 deacetylation, we conducted site prediction to identify potential acetylation sites. Sequence alignment across various species indicated high conservation of several residues within the NRF2 440-450AA and 505-519AA regions (Fig. 5A). We thus focused lysine site in these regions and constructed NRF2 KR mutants where lysine residues were replaced with arginine and compared acetylation levels of these KR mutants to wild type NRF2 (WT) in SIRT7 knockdown cells. The results indicated that NRF2 K443R and K518R mutants significantly abolished the increase of NRF2 acetylation caused by SIRT7 knockdown when compared with WT NRF2 (Fig. 5B). More importantly, K443R and K518R dramatically decreased NRF2 ubiquitination and strongly deceased NRF2/KEAP1 interaction (Fig. 5C, D). To further confirm the role of deacetylation of NRF2 at K443R and K518R on its stability and activation, we generated NRF2 K443, 518R (2KR) mutant and NRF2 K443, 518Q (2KQ) mutant which lysine residues were reconstituted with glutamine to mimic acetylation. 2KR mutant completely abolished the increase of NRF2 acetylation caused by SIRT7 knockdown when compared with WT (Fig. 5E). In addition, 2KR mutant significantly suppressed NRF2 ubiquitination and KEAP1 binding while 2KQ mutant even surpassed these effects when compared with WT (Fig. 5F, G). Overexpression of 2KR mutant significantly enhanced NRF2 target genes expression compared with WT while 2KQ mutant failed to produce this effect (Fig. 5H). We further examined intracellular localization and the results indicated that WT NRF2 predominantly localized in the cytoplasm, K518R and K443R slightly increased nuclear localization, and 2KR mutation significantly enhanced nuclear localization of NRF2 (Fig. 5I). Similarly, we found that Oxa increased nuclear localization of WT NRF2, which was completely abolished by 2KQ mutation (Fig. 5I). More importantly, we observed that compared to WT NRF2, 2KR mutant significantly reduced the fluorescence intensity of DHE and MitoSOX Red. The Oxa induced ROS accumulation was completely blocked by 2KR but not 2KQ mutates (Fig. 5J, K).

### SIRT7 deficiency exacerbated Oxa-induced liver injury in vivo

**Fig. 6:**
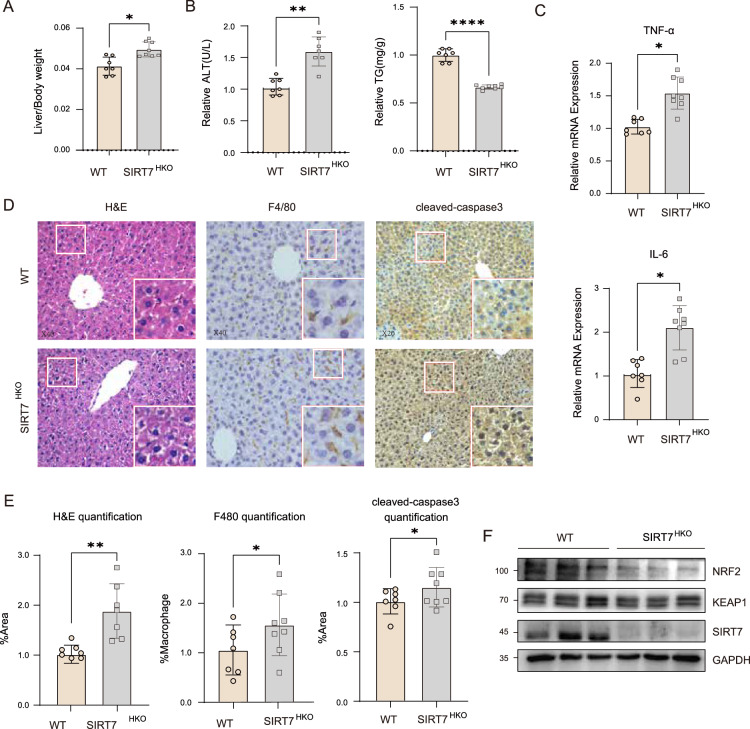
SIRT7 deficiency exacerbated Oxa-induced liver injury in vivo. **A** Quantification of the ratio of liver weight to the whole-body weight from WT and Hepatocyte specific SIRT7 knockout mice (*SIRT7*^*HKO*^) mice after Oxaliplatin treatment. **B** Serum ALT activity (left panel) and TG level (right panel) in WT and *SIRT7*^*HKO*^ mice after Oxaliplatin treatment. **C** Relative mRNA expression of proinflammatory cytokines TNF-α and IL-6 (using 2^−△△CT^ method; standardized to WT mice, after GAPDH normalization) in liver tissue of WT and *SIRT7*^*HKO*^ mice after Oxaliplatin treatment (*n* = 6–8 per group). Representative images of H&E staining, IHC staining for F4/80 and cleaved-caspase3 (**D**) as well as statistical quantification (**E**) were performed in liver sections of WT and *SIRT7*^*HKO*^ mice after Oxaliplatin treatment. **F** Protein level of NRF2/KEAP1 pathway were evaluated by western blotting in total liver homogenates from WT and *SIRT7*^*HKO*^ mice after Oxaliplatin treatment. Data derived from three independent experiments were presented as mean ± SEM. *p < 0.05, **p < 0.01, ***p < 0.001, ****p < 0.0001. ALT, alanine aminotransferase; TG, triglyceride; IHC, immunohistochemistry.

To evaluate the consequences of SIRT7 mediated NRF2 activation regulation, we generated hepatocyte specific *SIRT7* knockout mice (*SIRT7*^*HKO*^) by using albumin cre system and evaluated the role of SIRT7 in chemodrug Oxa-induced liver injury. Mice were treated with various concentrations of Oxaliplatin for 12 days, and the results indicated that both 6 mg/kg and 10 mg/kg Oxaliplatin caused high mortality in mice while 4 mg/kg Oxaliplatin mildly affected mice mortality, we thus chose this concentration for further investigation (Fig. S3A). Compared to the control group, mice in the Oxaliplatin group exhibited daily weight loss and increased liver-to-body weight ratio (Fig. S3B, S3C). More importantly, serum ALT in mice was significantly increased in the Oxaliplatin group, while liver TG levels were decreased significantly (Fig. S3D). In addition, RT-qPCR results showed that pro-inflammatory cytokines TNF-α and IL-6 were also enhanced by Oxaliplatin (Fig. S3E). H&E staining revealed that Oxaliplatin treatment induced hepatocyte nuclear shrinkage, sinusoidal dilation, and hepatocyte injury, which is associated with a remarkable increase of F4/80 positive cells and apoptosis-related marker cleaved-caspase3 (Fig. S3F). To investigate the potential role of SIRTs protein in Oxa-induced liver injury, we measured SIRTs protein expression in our mice and the results indicated Oxaliplatin treatment induced upregulation of SIRT7 while other SIRTs including SIRT3 and SIRT4 remain unchanged (Fig. S3G, H). In response to Oxa treatment, *SIRT7*^*HKO*^ mice showed a significant increase in liver-to-body weight ratio and serum ALT levels, while TG levels decreased significantly (Fig. 6A, B). TNF-α and IL-6 levels were also significantly enhanced in *SIRT7*^*HKO*^ mice (Fig. 6C). H&E and IHC staining results showed that compared to control mice, the degree of hepatocyte shrinkage and sinusoidal dilation, macrophage infiltration, and cleaved-caspased3 were also enhanced in *SIRT7*^*HKO*^ group (Fig. 6D, E). Consistently, we found that knockout of SIRT7 significantly impaired Oxa-induced NRF2 upregulation in vivo (Fig. 6F). Of note, we did not observe significant differences between control and *SIRT7*^*HKO*^ mice liver under normal condition (Fig. S4A), there were no significant differences in serum ALT and AST between two group of mice (Fig. S4B).

### Overexpression of NRF2 eliminated Oxa-induced liver injury in *SIRT7*^*HKO*^ mice

**Fig. 7:**
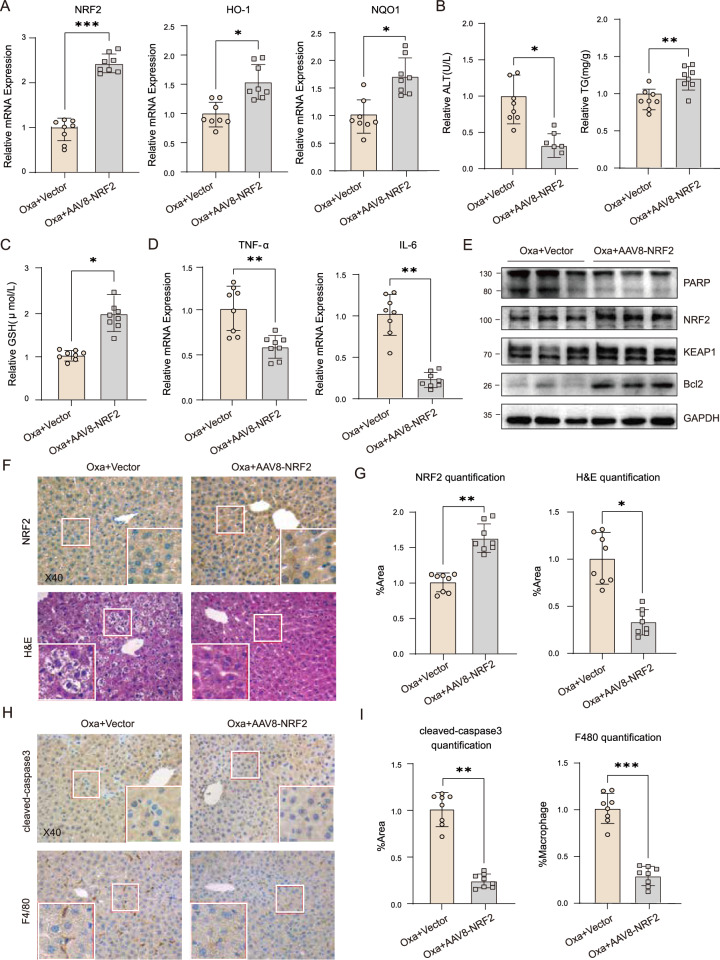
Overexpression of NRF2 eliminated Oxa-induced liver injury in *SIRT7*^*HKO*^ mice. **A** Relative mRNA expression of NRF2 and its target gene were determined by RT-qPCR in total liver homogenates from *SIRT7*^*HKO*^ mice infected with Adeno-associated virus (AAV8)-NRF2 vectors containing TBG promoter after Oxaliplatin treatment. Serum ALT activity, liver TG levels (**B**) and GSH levels (**C**) of the above *SIRT7*^*HKO*^ mice. **D** Relative mRNA expression of proinflammatory cytokines TNF-α and IL-6 (using 2^−△△CT^ method; standardized to *SIRT7*^*HKO*^ mice treated with control virus vectors, after GAPDH normalization) in liver tissue of *SIRT7*^*HKO*^ mice infected with Adeno-associated virus (AAV8)-NRF2 vectors containing TBG promoter after Oxaliplatin treatment (*n* = 6–8 per group). **E** Protein level of PARP, NRF2, KEAP1 and Bcl2 were determined by western blotting in total liver homogenates from mice described as above. Representative images of H&E staining, IHC staining for NRF2 (**F**), cleaved-caspase3 and F4/80 (**H**) as well as statistical quantification (**G**, **I**) were performed in liver sections of *SIRT7*^*HKO*^ mice. Data derived from three independent experiments were presented as mean ± SEM. *p < 0.05, **p < 0.01, ***p < 0.001, ****p < 0.0001.

To further confirm role of NRF2 for the protective effect of SIRT7 in vivo. We intravenously injected *SIRT7*^*HKO*^ mice with Adeno-associated virus (AAV8)-NRF2 vectors containing TBG promoter or control vectors one week before Oxa administration. We confirmed that AAV8-NRF2 vectors significantly enhanced NRF2 and its target genes expression in mice liver when compared with control vectors (Fig. 7A). After Oxa treatment, AAV8-NRF2 significantly decreased serum ALT levels, increased levels of TG and GSH, and reduced expression of pro-inflammatory cytokines TNF-α and IL-6 in *SIRT7*^*HKO*^ mice when compared to control vector (Fig. 7B–D). WB results indicated that AAV8-NRF2 significantly increased NRF2 protein expression, decreased PARP expression and increased Bcl2 expression in the mice liver when compared with control vectors (Fig. 7E). Histological examination revealed that restored NRF2 expression ameliorated degree of hepatocyte shrinkage and sinusoidal dilation, macrophage infiltration and cleaved-caspased 3 induced by Oxa treatment in *SIRT7*^*HKO*^ mice (Fig. 7F–I).

### Pharmacological activation of NRF2 alleviated Oxa-induced liver injury in *SIRT7*^*HKO*^ mice

**Fig. 8:**
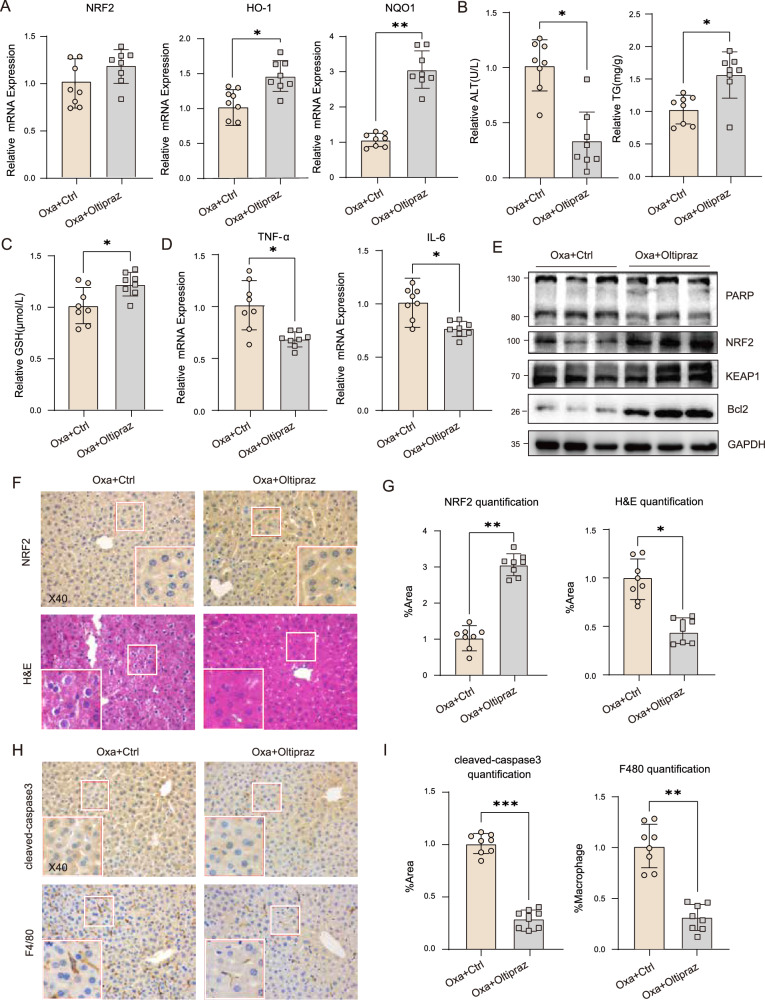
Pharmacological activation of NRF2 alleviated Oxa-induced liver injury in *SIRT7*^*HKO*^ mice. **A** Relative mRNA expression of NRF2 and its target genes in liver tissue of *SIRT7*^*HKO*^ mice. Serum ALT activity, liver TG level (**B**) and GSH levels (**C**) in *SIRT7*^*HKO*^ mice orally administered with Oltipraz after Oxaliplatin treatment. **D** Relative mRNA expression of proinflammatory cytokines TNF-α and IL-6 (using 2^−△△CT^ method; standardized to *SIRT7*^*HKO*^ mice orally administered with solvent, after GAPDH normalization) in liver tissue of *SIRT7*^*HKO*^ mice orally administered with Oltipraz after Oxaliplatin treatment (*n* = 6–8 per group). **E** Protein level of PARP, NRF2, KEAP1 and Bcl2 were determined by western blotting in total liver homogenates from mice described as above. Representative images of H&E staining, IHC staining for NRF2 (**F**), cleaved-caspase3 and F4/80 (**H**) as well as statistical quantification (**G**, **I**) were performed in liver sections of *SIRT7*^*HKO*^ mice. Data derived from three independent experiments were presented as mean ± SEM. *p < 0.05, **p < 0.01, ***p < 0.001, ****p < 0.0001.

Finally, to further validate the role of NRF2 in mediating the protective effect of SIRT7 in Oxa-induced liver injury, we treated mice with Oltipraz (OLT), a typical NRF2 agonist one week after Oxa administration. OLT markedly enhanced NRF2 target gene expression in the liver when compared with the control group (Fig. 8A). More importantly, we found that in Oxa treated *SIRT7*^*HKO*^ mice, OLT sufficiently decreased serum ALT, increased liver TG and GSH level, accompanied by reduction of pro-inflammatory cytokine including TNF-a and IL-6 expression (Fig. 8B–D). In addition, WB results indicated that OLT treatment decreased PARP and enhanced Bcl2 expression in *SIRT7*^*HKO*^ mice (Fig. 8E), and ameliorated the degree of hepatocyte shrinkage and sinusoidal dilation, macrophage infiltration, and cleaved-caspased 3 induced by Oxa treatment in *SIRT7*^*HKO*^ mice (Fig. 8F-I). Thus, our data provide novel translational insight by which SIRT7 and NRF2 biochemical crosstalk in regulating Oxa-induced liver injury.

## Discussion

In the present study, we reported that SIRT7 directly interacts with NRF2 and induces NRF2 deacetylation. Overexpression of SIRT7 significantly inhibited the binding of NRF2 to KEAP1, thereby enhancing its protein stability, facilitating its nuclear translocation and transactivation. While the enzymatically inactive mutant of SIRT7 abolished these effects. We further identified that SIRT7 induced NRF2 deacetylation at K443 and K518 sites. Lysine-arginine mutations of these sites (2KR NRF2) significantly reduced KEAP1 binding and ubiquitination, increased nuclear translocation and NRF2 target genes expression, whereas lysine-glutamine (2KQ NRF2) mutant showed similar subcellular localization and transactivation patterns with WT. Knockdown of SIRT7 led to a significant increase of apoptosis in response to oxidative stress in vitro and notably exacerbated Oxa-induced ALT elevation, hepatocellular damage, sinusoidal dilatation and inflammation in vivo. AAV8-NRF2-mediated hepatic NRF2 overexpression, along with NRF2 agonist treatment significantly reversed Oxa-induced elevation of ALT levels, hepatocellular damage, sinusoidal dilatation, and inflammation in *SIRT7*^*HKO*^ mice.

Oxa is the first-line drug widely used in advanced colorectal cancer patients but is often associated with hepatotoxicity [42, 43]. Studies have suggested that oxidative stress [44, 45] is involved in the regulation of Oxa-induced liver damage but the precise mechanism remains unclear. Studies have shown that ROS induced by Oxa aggravates hepatic oxidative stress, inflammation and hepatic fibrosis [46]. Administration of antioxidants including flavonoids rutin improves Oxa-induced peripheral neuropathy and liver injury in tumor-bearing mice model [47–49]. Consistent with these observations, we further identified that SIRT7 is critical in protecting against Oxa-induced liver injury via regulating NRF2, the key regulator responsible for ROS homeostasis. More importantly, we found that the NRF2 agonist Oltipraz significantly improved Oxa-induced hepatic injury in *SIRT7*^*HKO*^ mice. Our data thus clearly indicated the critical role of ROS homeostasis in Oxa-induced liver injury and suggested that targeting SIRT7-dependent NRF2 deacetylation could provide novel intervention options. Additionally, we found that the impact of SIRT7 on hepatocyte oxidative stress extends beyond Oxa to include various substances capable of inducing oxidative stress, such as APAP and H_2_O_2_. Whether SIRT7 participates in the regulation of the oxidative stress induced liver injury by these reagents would be of great interest.

NRF2 plays a key role in cell adaptation and survival under stress conditions by regulating mitochondrial function and orchestrating various networks of cell-protective proteins [50, 51]. In addition to KEAP1 mediated ubiquitin-proteasome degradation, post-translational modifications of NRF2 are critical for regulating its nuclear translocation and activation [52]. Studies have shown that acetylation is critical in regulating NRF2 nuclear translocation but the precise mechanism has yet to be determined. A study shows that SIRT6 deacetylates and stabilizes NRF2 to increase the expression of anti-oxidative genes and defend against ROS overload [16]. Knockdown of SIRT6 significantly inhibited APAP-induced phosphorylation of NRF2, while the phosphorylation of NRF2 facilitates its release from KEAP1 in response to oxidative stress, stabilizing NRF2 and allowing it to translocate to the nucleus [53]. On the other hand, SIRT1 overexpression in cardiomyocytes or whole hearts significantly decreases the acetylation of NRF2, leading to the upregulation of its downstream signaling pathway [54]. These data clearly indicated the complexity of acetylation in determining NRF2 nuclear localization and activation. We observed that SIRT7 directly binds to NRF2 and induces its deacetylation at lysine 443 and 518. By mutating these lysine (K) residues to arginine (R) and substituting suspected sites from lysine to glutamine (Q), we demonstrated that lysine 443 and 518 are critical for NRF2 stabilization, nuclear localization and activation. More importantly, 2KR NRF2 mutant significantly suppressed NRF2 ubiquitination and KEAP1 binding while 2KQ mutant even surpassed these effects. Our data thus provided evidence that acetylation at specific sites might determine NRF2 stability and nuclear localization via interrupting NRF2/KEAP1 interaction.

SIRT7 is involved in the control of oxidative stress through the regulation of ROS production [33] and mitochondrial function [27]. Knockout SIRT7 in mice results in multiple defects as consequence of mitochondrial dysfunction. ROS disturbs multiple biological processes including cell metabolism, aging, and cell death via damaging cell components such as DNA, RNA, and lipids [55–57]. In addition, SIRT7 modulates histone H3K18Ac which is critical in recruiting damage response factor 53BP1 and influences of DNA repair and cell death [58–60]. Consistent with this notion, we observed that knockout of SIRT7 increased chemodrug induced ROS accumulation and cell death. Downstream targets responsible for SIRT7 mediated ROS regulation are currently unclear but we identified NRF2 as a crucial target that participates in ROS homeostasis and cell survival. By using an in vivo mouse model, we have demonstrated that SIRT7/NRF2 is crucial for protecting against liver injury induced by chemodrug. More importantly, overexpression of NRF2 or NRF2 agonists sufficiently ameliorated chemodrug-induced liver injury in *SIRT7*^*HKO*^ mice. In addition, SIRT7 deacetylates USP39 to promote its protein stability and enhance autophagy, while inhibiting ROS production in CSCC cells [33]. In consistent with these observations, we found that knockdown of SIRT7 resulted in impairment of autophagy, but active autophagy by rapamycin had no effects on SIRT7 mediated chemosensitivity. Our data thus clearly illustrated the critical role of NRF2 in SIRT7 mediated antioxidative response and also extended our knowledge of SIRT7’s biological functions.

In conclusion, our data revealed previously unidentified roles of SIRT7 in modulating NRF2 nuclear localization and activation through deacetylation, thereby protecting against chemodrug-induced liver injury. Activating SIRT7 could provide a potential strategy for mitigating liver damage caused by chemotherapy.

## Supplementary information

Supporting information

Western Blots

## Data Availability

All data are within the manuscript and supporting information. Any additional information or data is available upon request.
