# Supplementary material for: SIRT7-mediated NRF2 deacetylation promotes antioxidant response and protects against chemodrug-induced liver injury
Source: Cell Death Dis. 2025 Apr 1;16(1):232. doi: 10.1038/s41419-025-07549-5 (PMC11961749; doi:10.1038/s41419-025-07549-5)
Supplement: Supplementary file 1 — Supporting information [file 41419_2025_7549_MOESM1_ESM.docx]

**Supporting Information for Original article**

**SIRT7-mediated NRF2 deacetylation promotes antioxidant response and protects against chemodrug-induced liver injury**

Tingzi Yu^1^, Cong Ding^1^, Jinying Peng^1^, Gaoshuang Liang^1^, Yongyi Tang^1^, Jinqiu Zhao^2,3#^ and Zhuan Li^1#^

^1^The Key Laboratory of Study and Discovery of Small Targeted Molecules of Hunan Province, The Key Laboratory of Model Animals and Stem Cell Biology of Hunan Province, Engineering Research Center of Reproduction and Translational Medicine of Hunan Province, and Institute of Interdisciplinary Studies, Hunan Normal University School of Medicine, Changsha, Hunan, 410013 China;

^2^Department of infectious disease, The First Affiliated Hospital of Chongqing Medical University, Chongqing, 400016, China;

^3^Department of Cancer center, University of Hawaii at Manoa, Honolulu, Hawaii,96813, USA;

**Supplementary Figure 1**

**
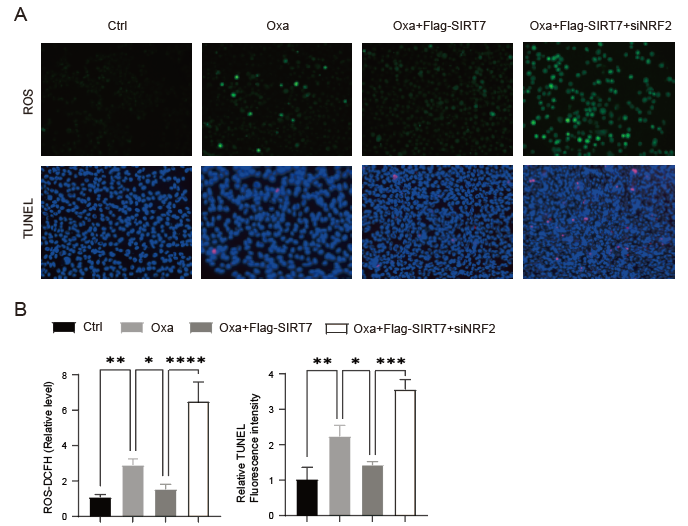
**

**Figure. S1.** (A) Huh7 cells were first transfected with siRNA targeting NRF2. 24 hours after siRNA transfection, Flag-tagged SIRT7 plasmid was transfected. Cells were then treated with 15 μM of Oxaliplatin for 24 hours before harvest. Representative images of intercellular ROS and apoptosis levels from each group were shown. (B) Quantitative analysis of relative fluorescence intensity in (A). Data derived from three independent experiments were presented as mean ± SEM. *p < 0.05, **p < 0.01, ***p < 0.001, ****p < 0.0001.

**Supplementary Figure 2**

**
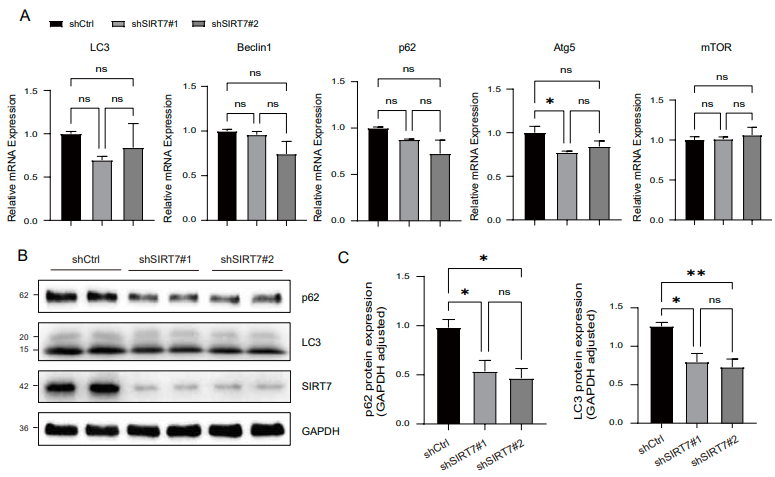
**

**
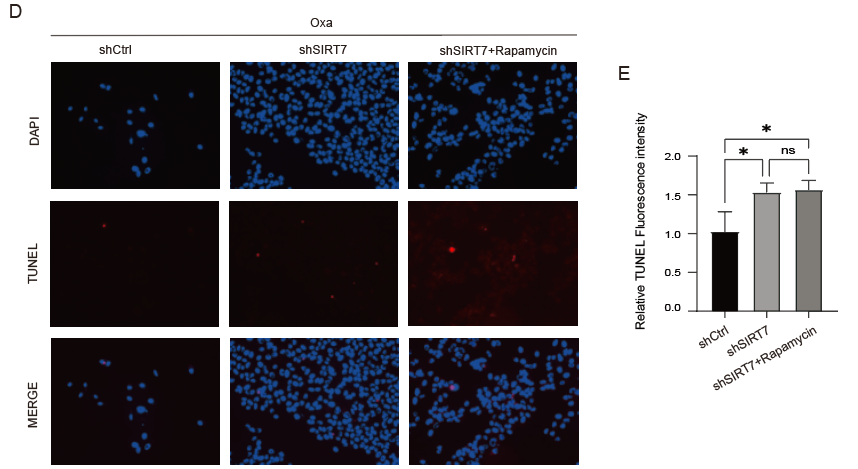
**

**Figure. S2.** (A) Relative mRNA expression of autophagy-related genes was determined by RT-qPCR in WT and SIRT7-KD Huh7 cells. (B) Protein level of p62 and LC3 were determined by western blotting in WT and SIRT7-KD Huh7 cells. (C) Quantitative analysis of p62 and LC3 panels in (B). (D) SIRT7-KD Huh7 cells were treated with Rapamycin 50uM for 12h, and then incubated with Oxaliplatin 15 uM for 24 h. Apoptosis levels were evaluated using the TUNEL assay, and representative images from each group were shown. Red fluorescence indicated TUNEL-positive apoptotic cells, and blue fluorescence represented DAPI-stained nuclei. (E) Quantitative analysis of relative fluorescence intensity in (D). Data derived from three independent experiments were presented as mean ± SEM. *p < 0.05, **p < 0.01, ***p < 0.001, ****p < 0.0001.

**Supplementary Figure 3**

**
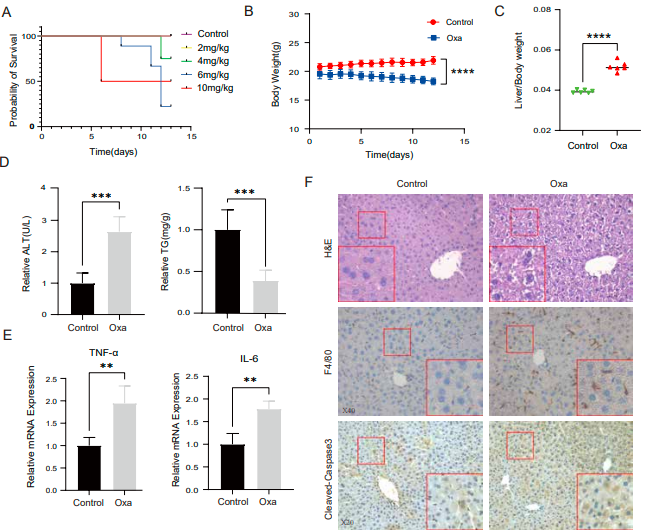

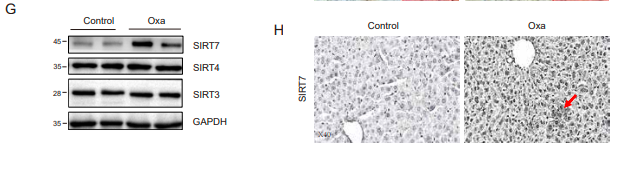
**

**Figure. S3. Oxaliplatin treatment resulted in elevation of SIRT7 and liver injury.** (A) Survival curves of C57BL/6 mice treated with different doses of Oxaliplatin. (B, C) Body Weight changes (B) and quantification of the ratio of liver weight to the whole-body weight (C) from C57BL/6 mice treated with solvent or Oxaliplatin 4mg/kg for 12 days. (D) Serum ALT activity (left panel) and TG level (right panel) in mice receiving indicated treatments. (E) Relative mRNA expression of proinflammatory cytokines TNF-α and IL-6 (using 2^−△△CT^ method; standardized to solvent-treated controls, after GAPDH normalization) in liver tissue of mice receiving indicated treatments (n = 6-8 per group). (F) Representative images of H&E staining and IHC staining for F4/80 and cleaved-caspase3 in mice liver section. (G) Protein level of SIRT3, SIRT4 and SIRT7 were determined by western blotting in total liver homogenates. (H) Representative images of IHC staining for SIRT7(Proteintech, #12994-1-AP) in mice liver section. Data derived from three independent experiments were presented as mean ± SEM. **p* < 0.05, ***p* < 0.01, ****p* < 0.001, *****p* < 0.0001.

**Supplementary Figure 4**


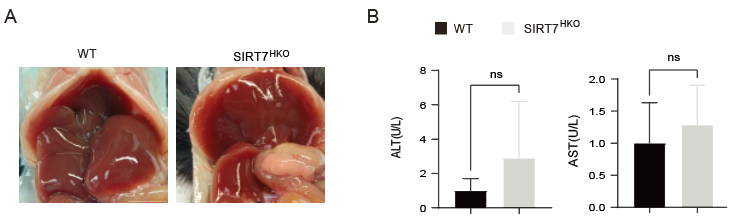


**Figure. S4.** (A) WT and *SIRT7 ^HKO^* mice were untreated, pictures were taken at the sacrificed time point (5 mice for each group; Scale bar, 1 cm). (B) Serum ALT activity (left panel) and AST activity (right panel) in WT and *SIRT7 ^HKO^* mice without any treatment.
