# Supplementary material for: SIRT7-mediated NRF2 deacetylation promotes antioxidant response and protects against chemodrug-induced liver injury
Source: Cell Death Dis. 2025 Apr 1;16(1):232. doi: 10.1038/s41419-025-07549-5 (PMC11961749; doi:10.1038/s41419-025-07549-5)

Fig 1A

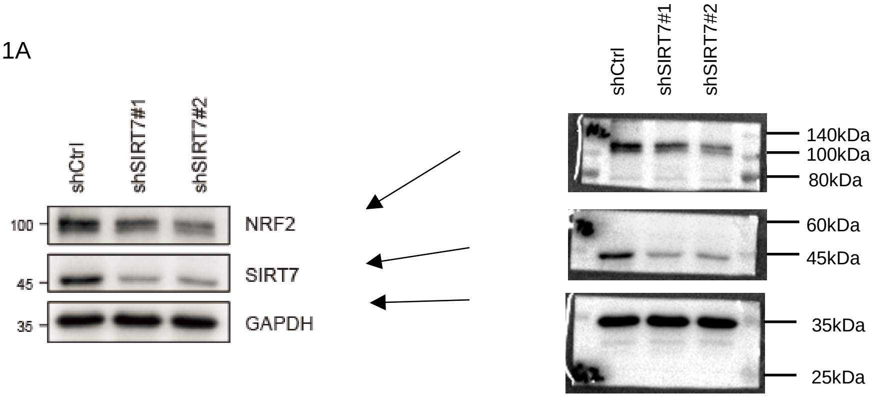

Fig 1B

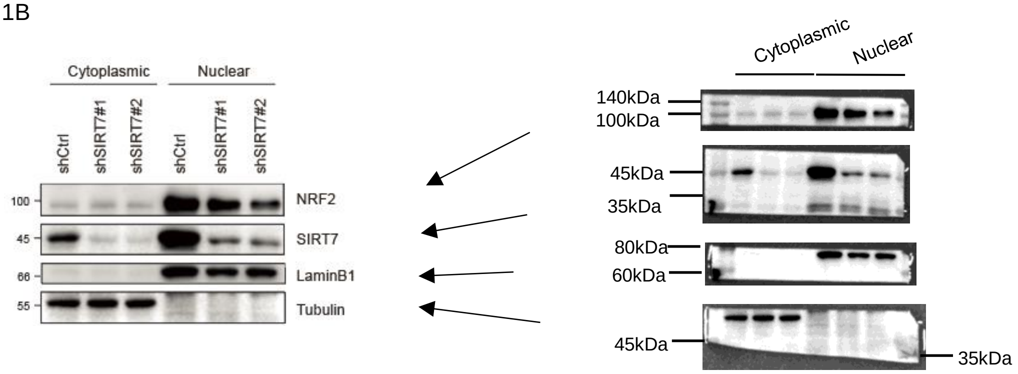

Fig 1E

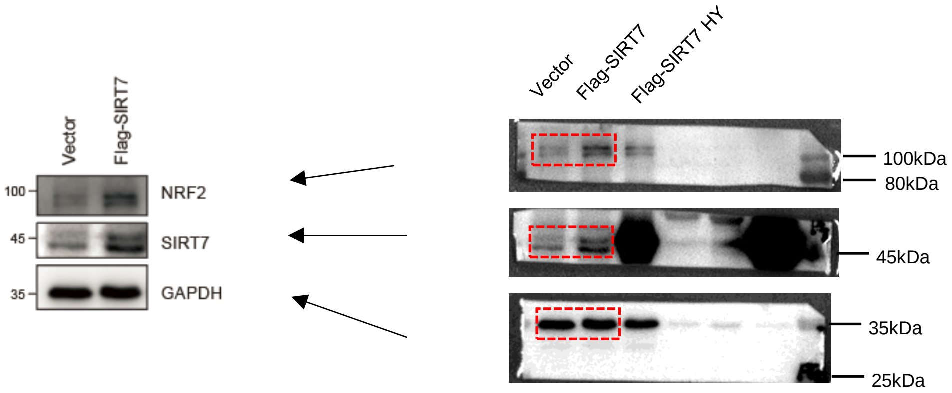

Fig 1F

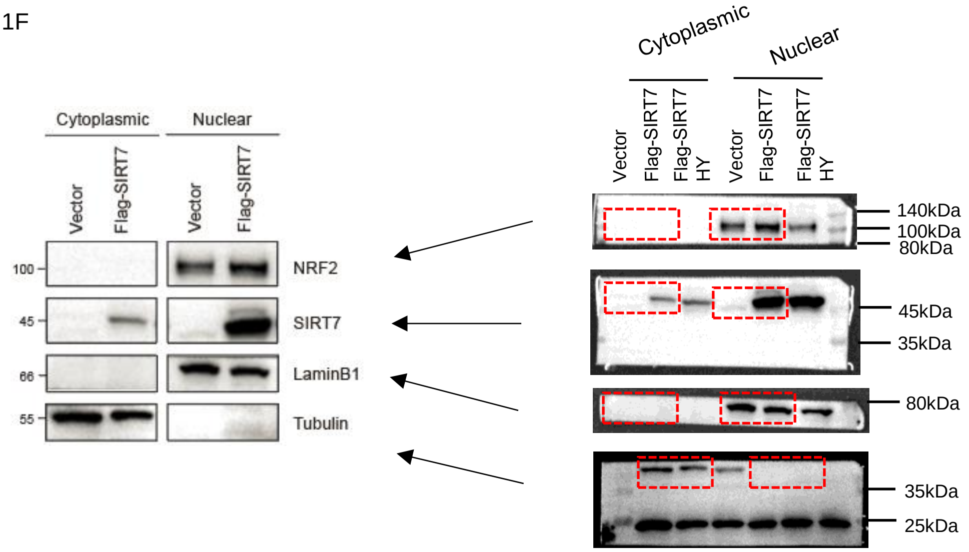

Fig 2F

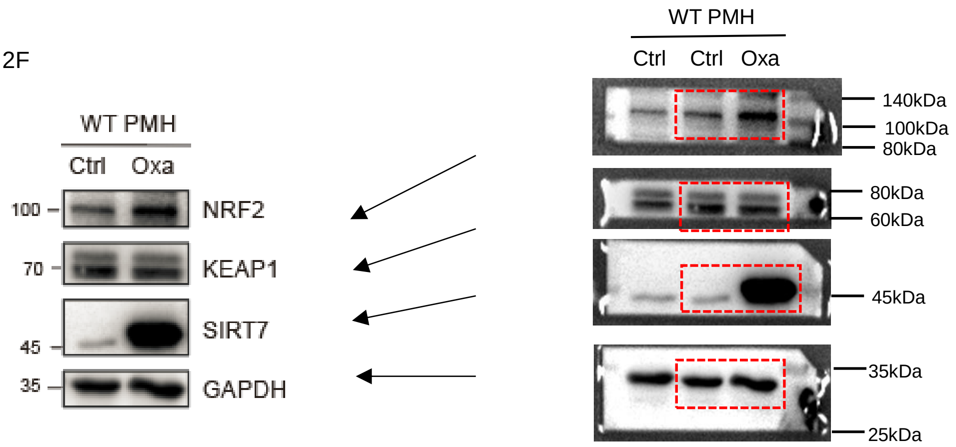

Fig 2H

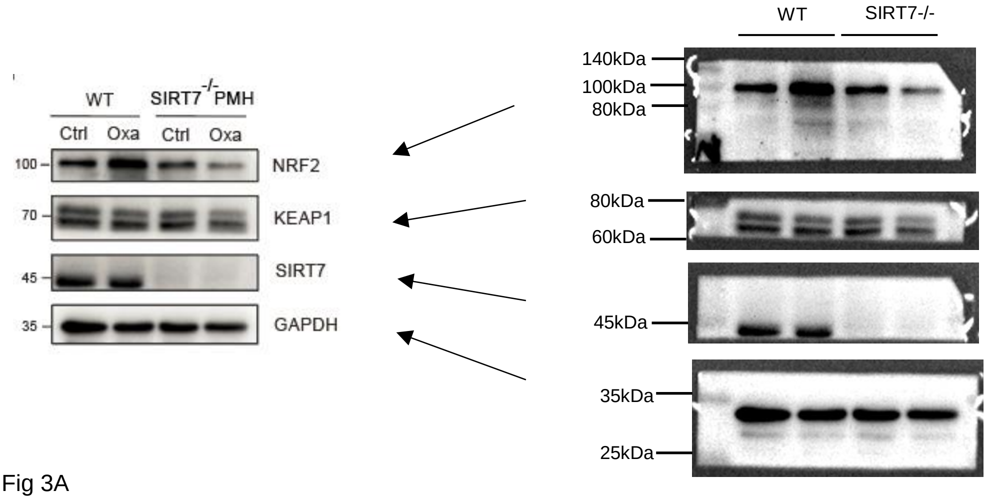

Fig 3A

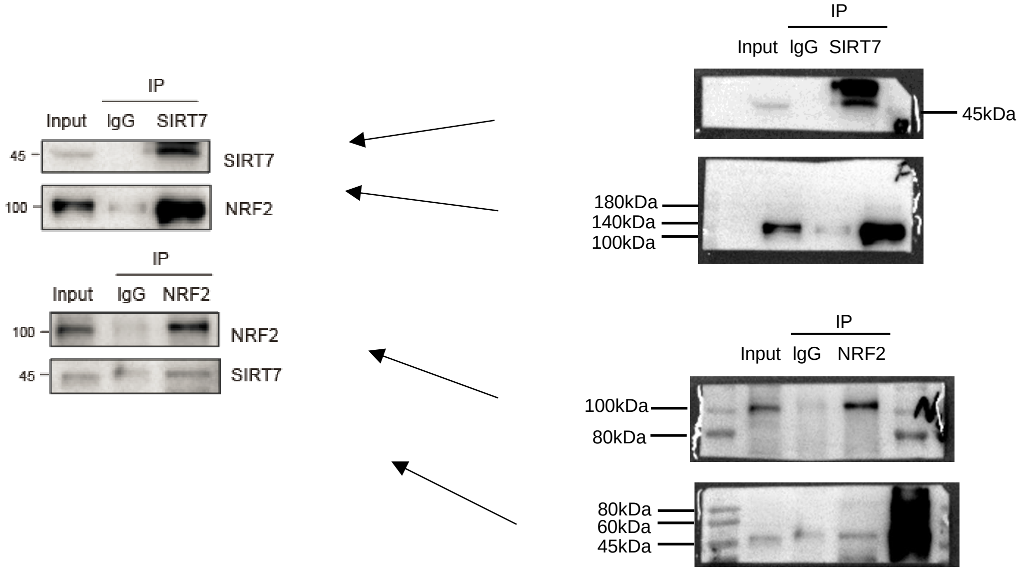

Fig 3B

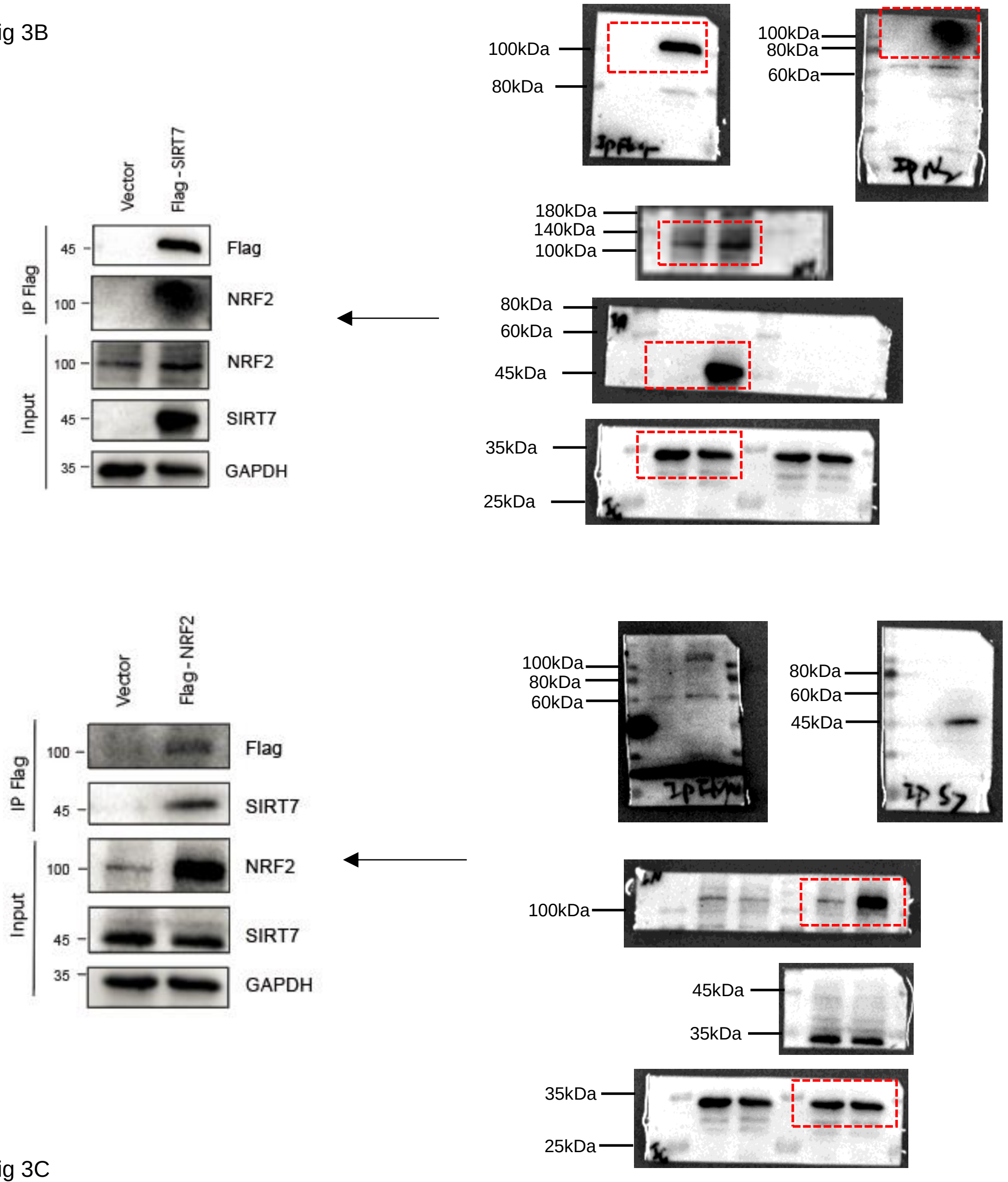

Vector
Flag - SIRT7
IP Flag
Input
45
100
100
45
35
Flag
NRF2
NRF2
SIRT7
GAPDH
100kDa
80kDa
100kDa
80kDa
60kDa
180kDa
140kDa
100kDa
80kDa
60kDa
45kDa
35kDa
25kDa
Vector
Flag - NRF2
IP Flag
Input
100
45
100
45
35
Flag
SIRT7
NRF2
SIRT7
GAPDH
100kDa
80kDa
60kDa
80kDa
60kDa
45kDa
100kDa
45kDa
35kDa
35kDa
25kDa

Fig 3C

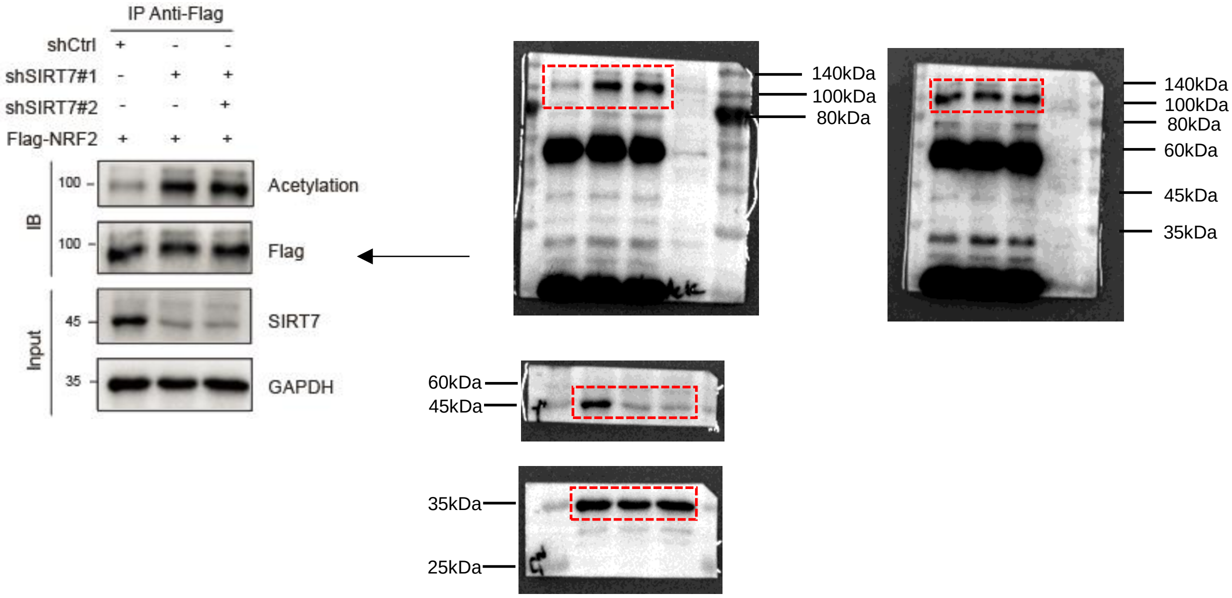

IP Anti-Flag
shCtrl + - -
shSIRT7#1 - + +
shSIRT7#2 - - +
Flag-NRF2 + + +
IB
Input
100
100
45
35
Acetylation
Flag
SIRT7
GAPDH
140kDa
100kDa
80kDa
140kDa
100kDa
80kDa
60kDa
45kDa
35kDa
60kDa
45kDa
35kDa
25kDa

Fig 3D

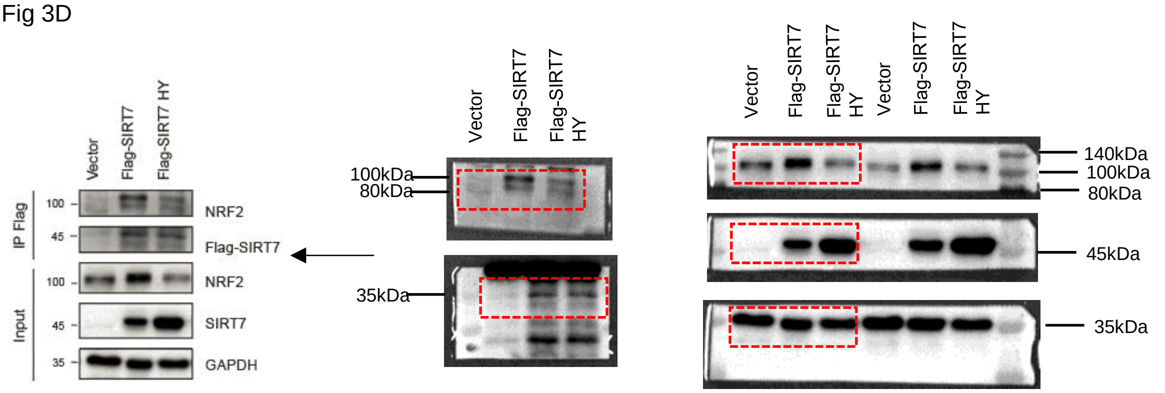

Fig 3E

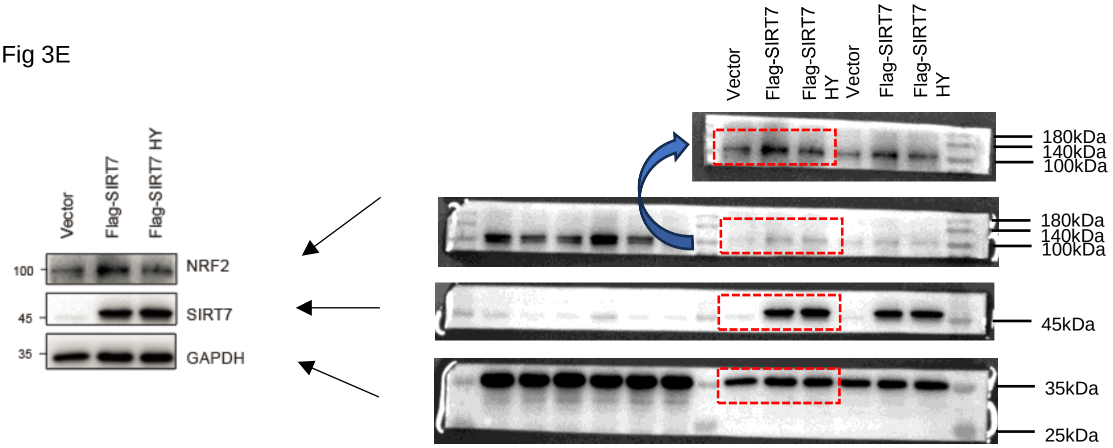

Fig 4A

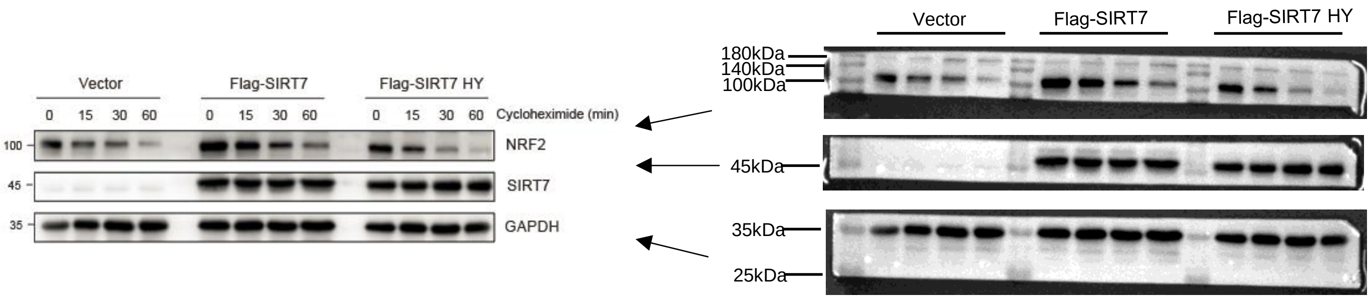

Fig 4C

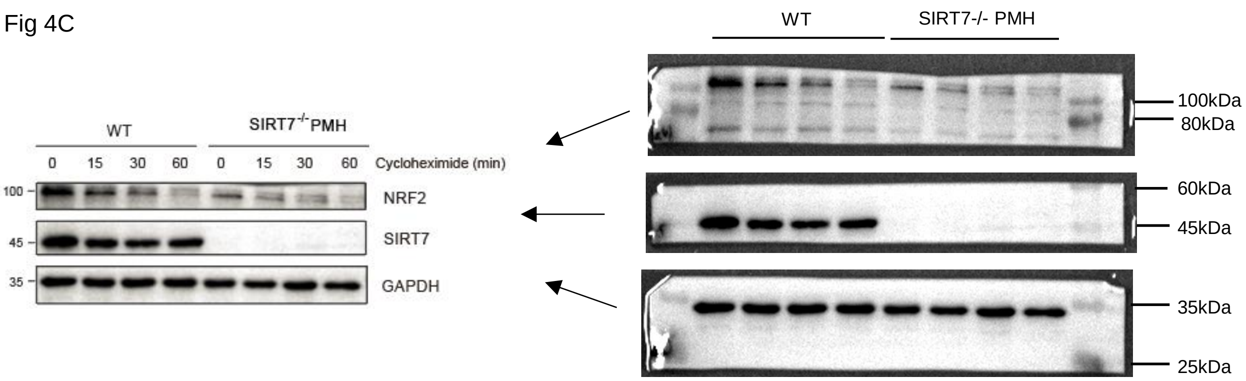

Fig 4E

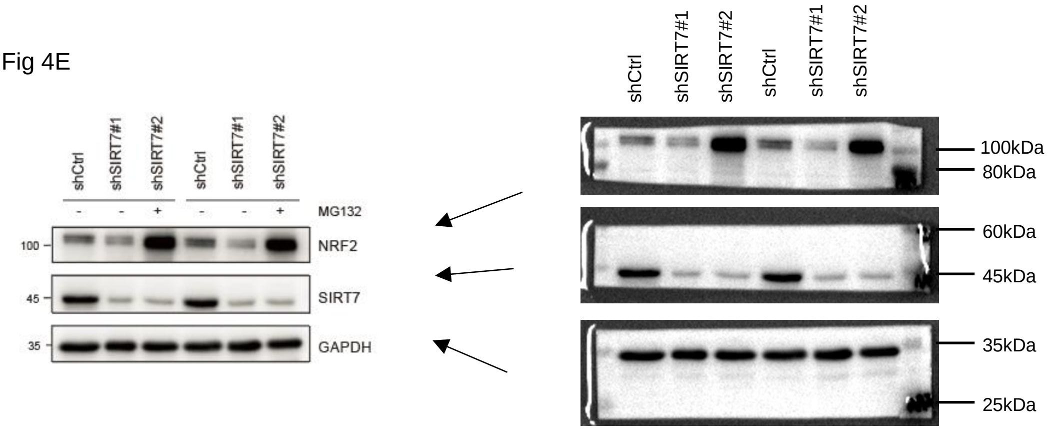

Fig 4F

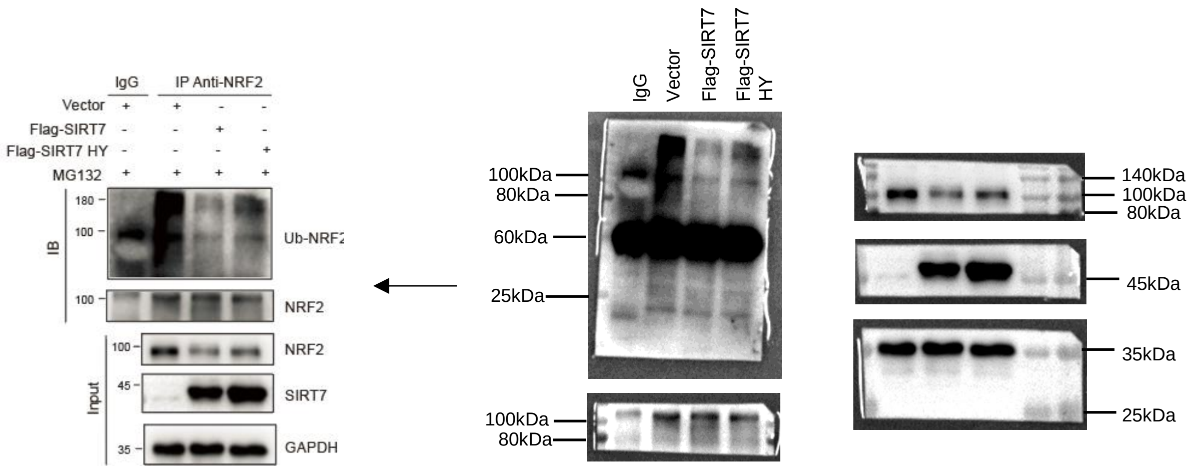

Fig 4G

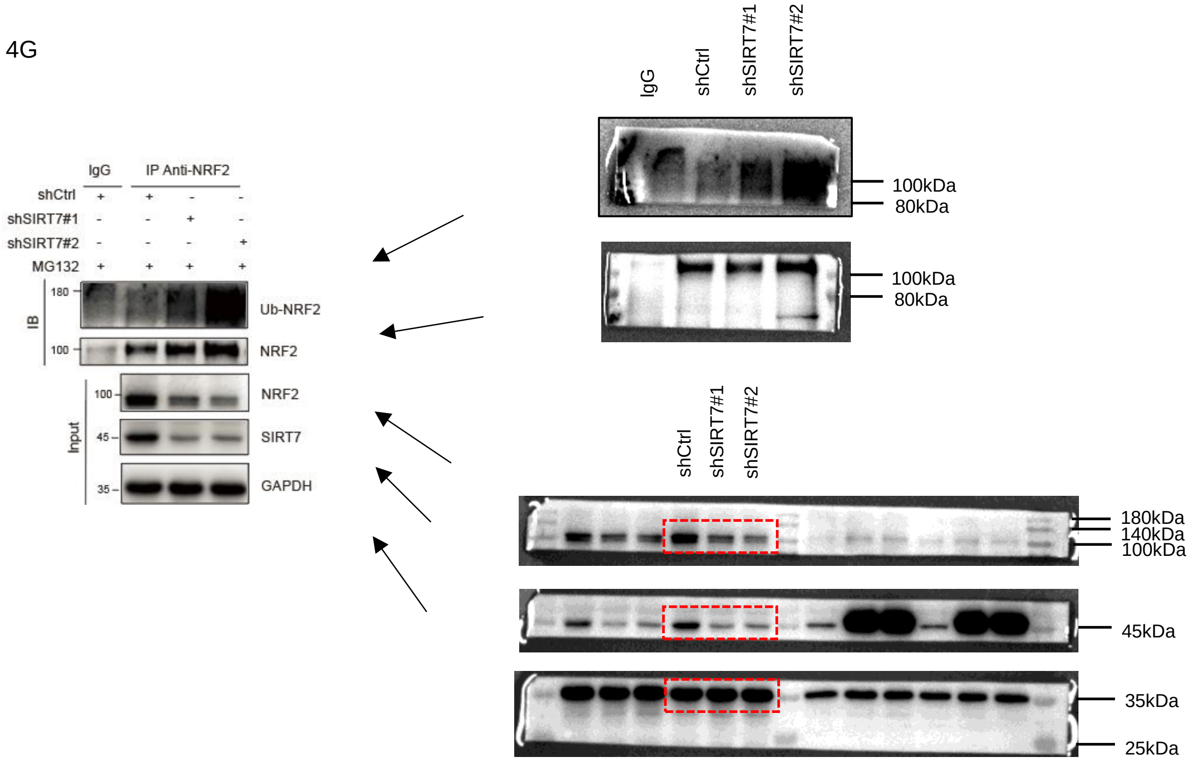

Fig 4H

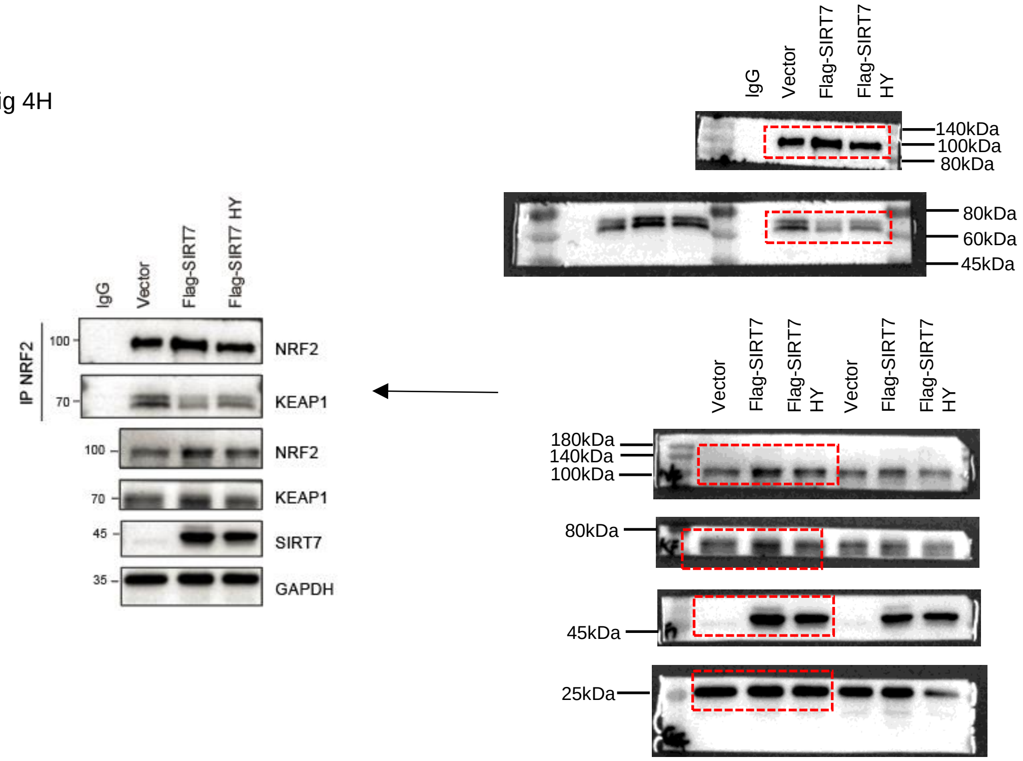
IgG
Vector
Flag-SIRT7
Flag-SIRT7 HY
IP NRF2
100
70
45
35
NRF2
KEAP1
NRF2
KEAP1
SIRT7
GAPDH
140kDa
100kDa
80kDa
80kDa
60kDa
45kDa
Vector
Flag-SIRT7
Flag-SIRT7 HY
Vector
Flag-SIRT7
Flag-SIRT7 HY
180kDa
140kDa
100kDa
80kDa
45kDa
25kDa

Fig 4I

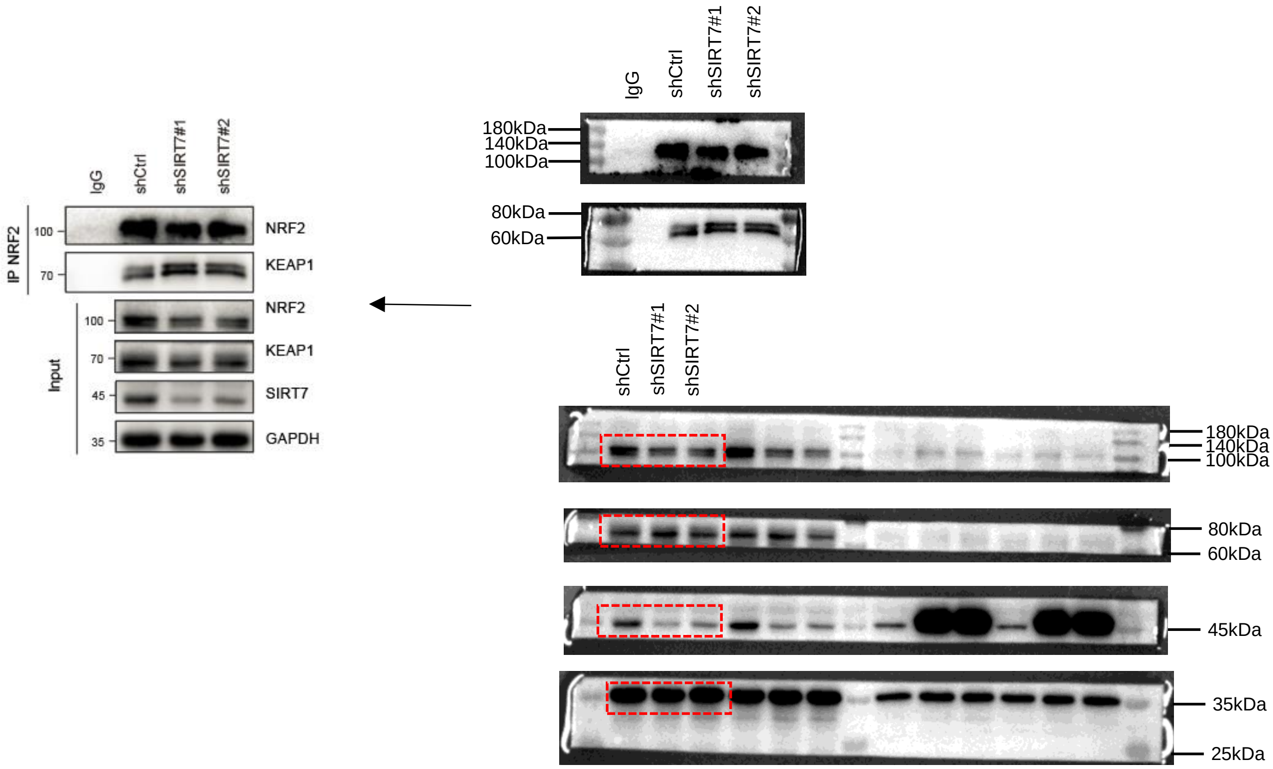
IgG
shCtrl
shSIRT7#1
shSIRT7#2
IP NRF2
Input
100
70
100
70
45
35
NRF2
KEAP1
NRF2
KEAP1
SIRT7
GAPDH
180kDa
140kDa
100kDa
80kDa
60kDa
shCtrl
shSIRT7#1
shSIRT7#2
180kDa
140kDa
100kDa
80kDa
60kDa
45kDa
35kDa
25kDa

Fig 5B

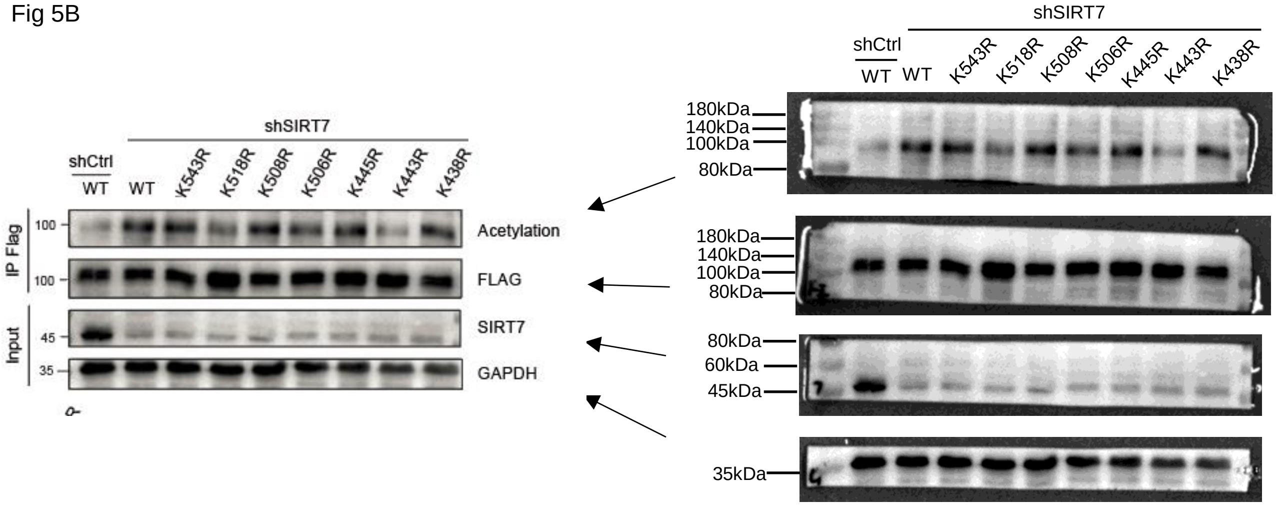

Fig 5C

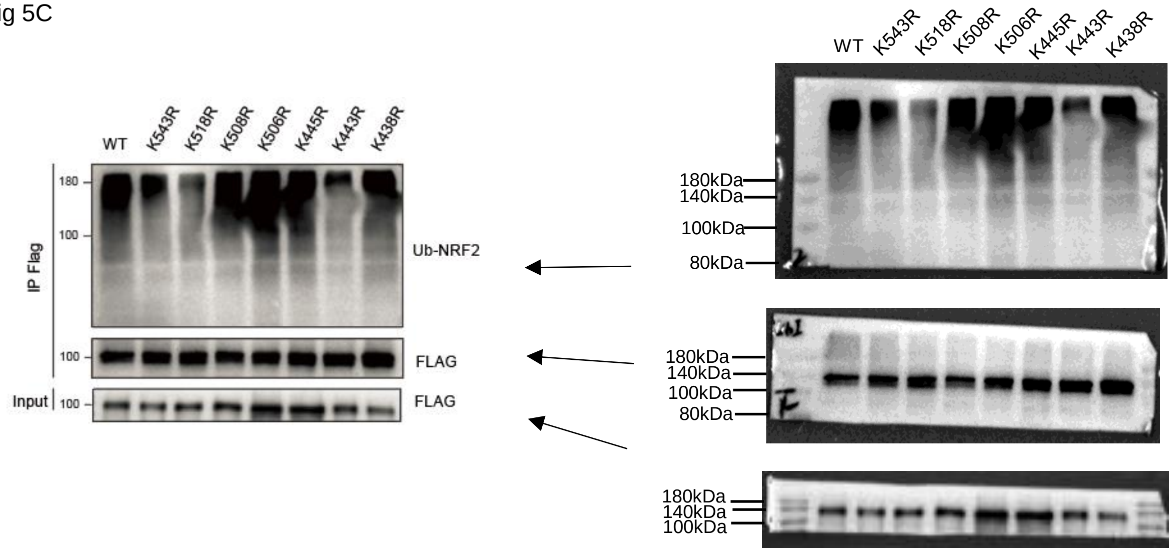

Fig 5D

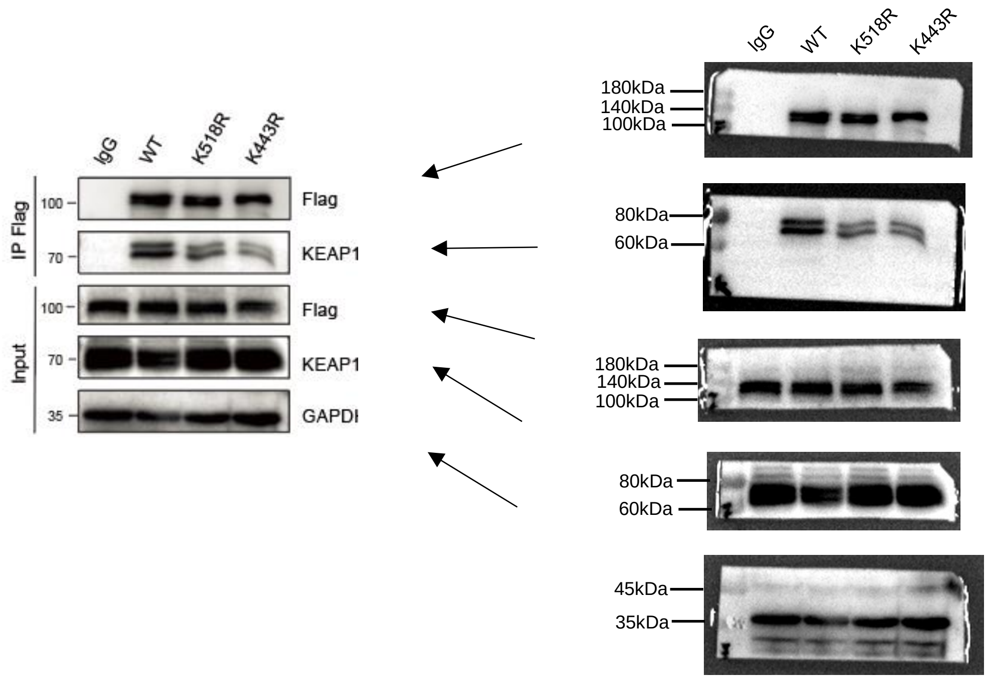

Fig 5E

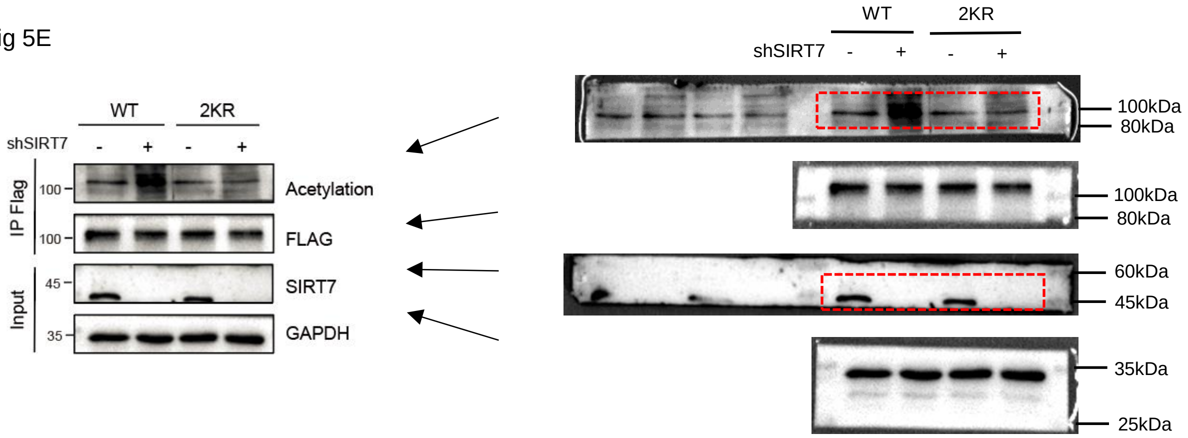

Fig 5F

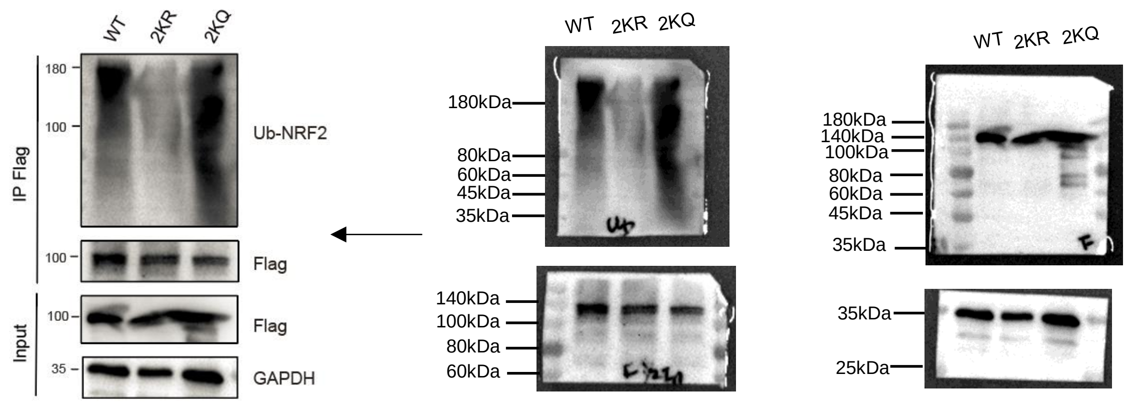

Fig 5G

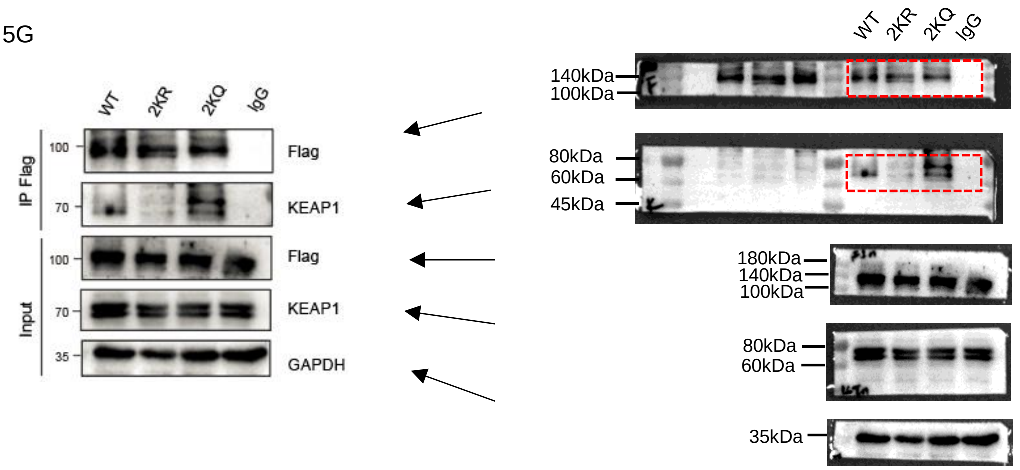

Fig 6E

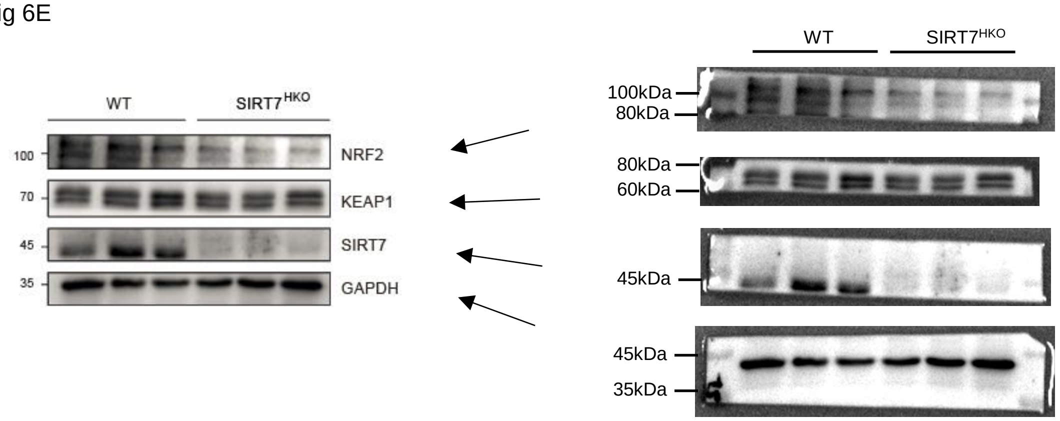

Fig 7E

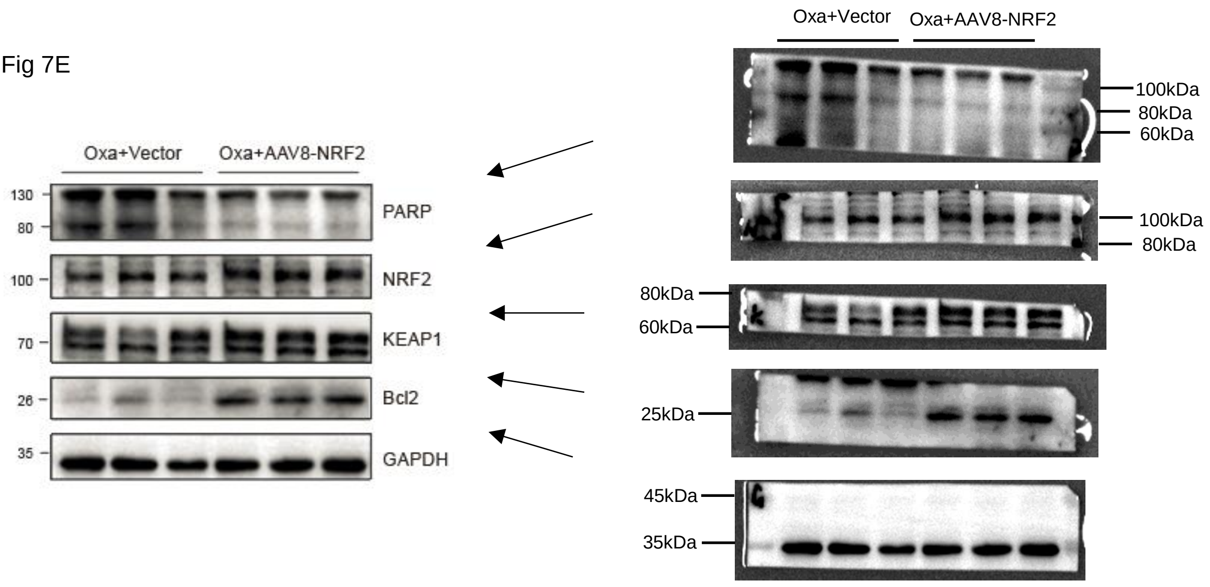
Oxa+Vector
Oxa+AAV8-NRF2
130
80
PARP
100
NRF2
70
KEAP1
26
Bcl2
35
GAPDH
Oxa+Vector
Oxa+AAV8-NRF2
100kDa
80kDa
60kDa
100kDa
80kDa
80kDa
60kDa
25kDa
45kDa
35kDa

Fig 8E

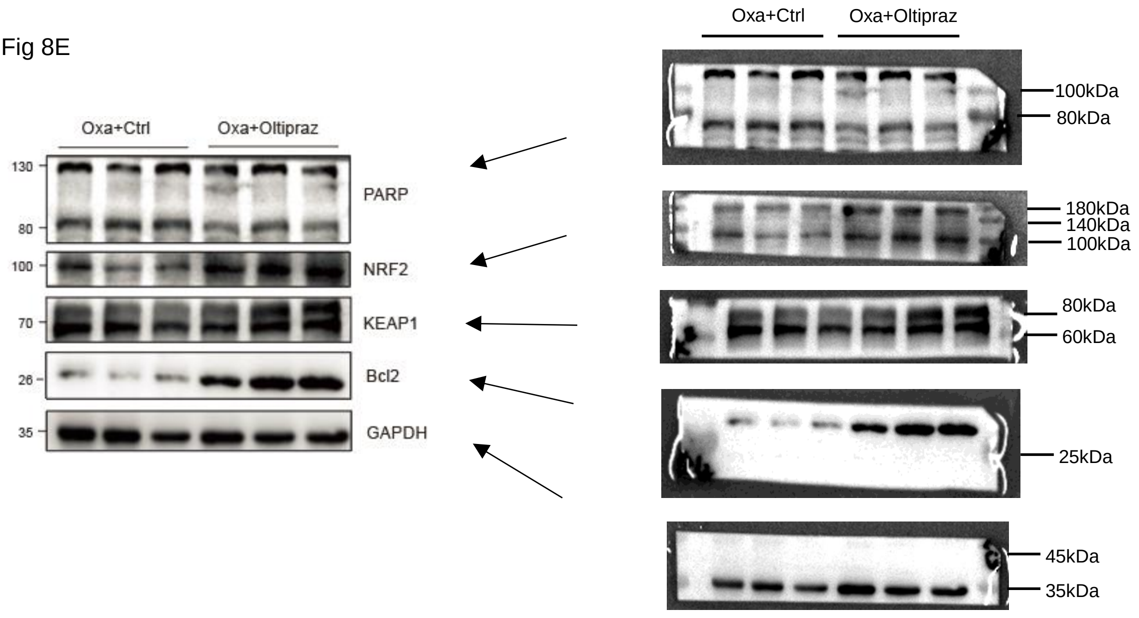
Oxa+Ctrl
Oxa+Oltipraz
130
80
PARP
100
NRF2
70
KEAP1
26
Bcl2
35
GAPDH
Oxa+Ctrl
Oxa+Oltipraz
100kDa
80kDa
180kDa
140kDa
100kDa
80kDa
60kDa
25kDa
45kDa
35kDa

Fig S2B

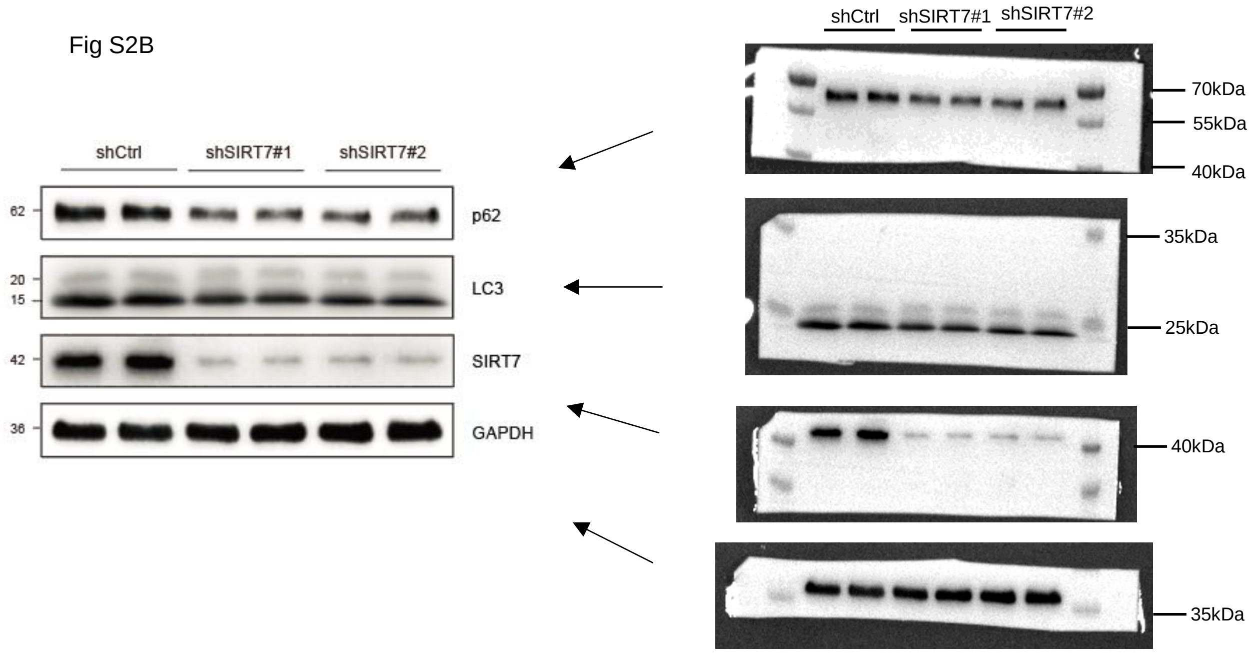

Fig S3G

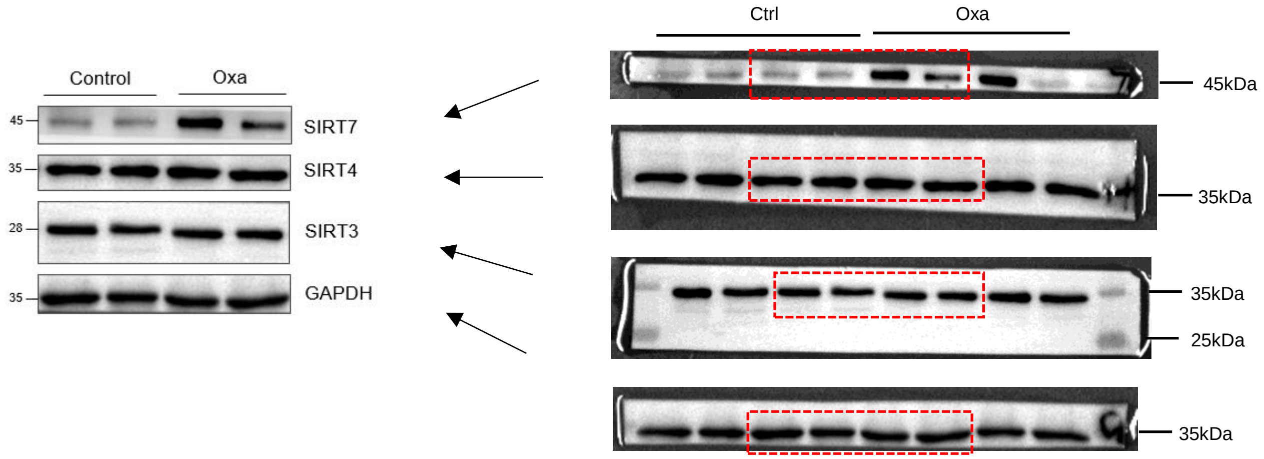

Supplement: Supplementary file 2 — Western Blots [file 41419_2025_7549_MOESM2_ESM.pdf]
